# Supplementary material for: Transposable elements contribute to cell and species-specific chromatin looping and gene regulation in mammalian genomes
Source: Nat Commun. 2020 Apr 14;11:1796. doi: 10.1038/s41467-020-15520-5 (PMC7156512; doi:10.1038/s41467-020-15520-5)
Supplement: Supplementary file 1 — Supplementary Information [file 41467_2020_15520_MOESM1_ESM.pdf]

Supplementary Figures and Tables

Transposable elements contribute to cell and species-specific chromatin looping and gene regulation in mammalian genomes.

Adam G Diehl, Ningxin Ouyang, Alan P Boyle

Table of Contents

Supplementary Table 1. Results for binomial tests of CTCF enrichment in human and mouse transposable elements.
Supplementary Table 2. Results for permutation tests of CTCF enrichment in human and mouse transposable elements.
Supplementary Table 3. Results for word-enrichment tests of CTCF motif enrichment in human and mouse transposable elements.
Supplementary Fig. 1: CTCF enrichments are robust to different statistical testing procedures and stable across cell types.
Supplementary Table 4. Enriched motif-words in CTCF motif-word enrichment tests in human and mouse transposable elements.
Supplementary Fig. 2: Differences in functional annotation associations do not explain CTCFbinding enrichments in human and mouse TE types.
Supplementary Fig. 3: TE-loop associations are stable across species and cell lines.
Supplementary Table 5. Motif orientations within TE-loop intersections in human and mouse cells.
Supplementary Fig. 4: Comparison of histone modifications within TE-derived vs non-TE-derived loop anchors.
Supplementary Fig. 5: Conservation class assignment algorithm.
Supplementary Fig. 6: Conservation classes for all pairwise cell comparisons.
Supplementary Fig. 7: Loop comparison resolution has minmal effects on conservation class membership.
Supplementary Fig. 8: Overlaps between loops in the present dataset and known TADs and TAD boundaries.
Supplementary Fig. 9: All conservation classes of TE-derived loops show evidence of natural selection.
Supplementary Fig. 10: Comparison of histone modifications for non-conserved loops.
Supplementary Fig. 11: Associations between transposable elements and conservation classes are stable across cell lines.
Supplementary Table 6. Contribution of TEs to loop conservation classes.
Supplementary Fig. 12: Contributions of transposable elements to conserved and non-conserved chromatin loops are not correlated with loop comparison resolution.
Supplementary Fig. 13: TE-derived variable chromatin loops are significantly associated with variable gene expression.

Supplementary Table 1: CTCF-enriched transposable element types from binomial test procedure

				GM12878				K562				CH12				MEL			
	**TE Type**	**TE Class**	**TE Family**	**# Insertions**	**# Bound**	**% Bound**	**p-value**	**# Insertions**	**# Bound**	**% Bound**	**p-value**	**# Insertions**	**# Bound**	**% Bound**	**p-value**	**# Insertions**	**# Bound**	**% Bound**	**p-value**
**Shared**	**MER20**	DNA	hAT-Charlie	16570	587	3.5%	< 2E-200	16570	602	3.6%	< 2E-200	5989	171	2.9%	4.1E-32	5989	140	2.3%	2.9E-21
	**LTR41**	LTR	ERVL	1111	134	12.1%	5.5E-170	1111	138	12.4%	1.2E-159	499	62	12.4%	4.6E-45	499	60	12.0%	1.1E-44
	**LTR41B**	LTR	ERVL	832	111	13.3%	9.9E-146	832	109	13.1%	1.7E-128	287	47	16.4%	1.4E-39	287	50	17.4%	2.9E-45
	**MER91B**	DNA	hAT-Tip100	1406	113	8.0%	9.9E-123	1406	119	8.5%	2.9E-117	232	35	15.1%	7.0E-28	232	33	14.2%	1.9E-26
	**MamGypLTR3**	LTR	Gypsy	660	55	8.3%	4.9E-60	660	53	8.0%	2.2E-50	115	17	14.8%	1.1E-12	115	17	14.8%	3.0E-13
	**LTR50**	LTR	ERVL	2547	185	7.3%	2.2E-193	2547	199	7.8%	7.6E-190	547	24	4.4%	8.7E-07	547	19	3.5%	4.6E-04
	**MER91C**	DNA	hAT-Tip100	1616	104	6.4%	8.1E-103	1616	130	8.0%	2.5E-125	105	9	8.6%	6.2E-04	105	8	7.6%	3.4E-03
	**LTR55**	LTR	ERV	406	53	13.1%	1.9E-68	406	50	12.3%	6.4E-57	150	10	6.7%	1.6E-03	150	11	7.3%	8.5E-05
**Human-Specific**	**LTR13**	LTR	ERVK	455	203	44.6%	< 2E200	455	253	55.6%	< 2E200	0	--	--	--	0	--	--	--
	**HERVK-int**	LTR	ERVK	244	41	16.8%	1.9E-57	244	44	18.0%	1.2E-57	0	--	--	--	0	--	--	--
	**LTR41C**	LTR	ERVL	746	84	11.3%	1.5E-103	746	78	10.5%	9.6E-84	0	--	--	--	0	--	--	--
	**L1PA15-16**	LINE	L1	1365	93	6.8%	4.0E-94	1365	82	6.0%	2.2E-68	0	--	--	--	0	--	--	--
	**HUERS-P3-int**	LTR	ERV1	663	60	9.0%	8.8E-68	663	73	11.0%	5.5E-80	0	--	--	--	0	--	--	--
	**HERVE-int**	LTR	ERV1	267	37	13.9%	2.0E-48	267	30	11.2%	2.1E-32	0	--	--	--	0	--	--	--
	**LTR13_**	LTR	ERVK	35	20	57.1%	4.0E-40	35	25	71.4%	4.0E-51	0	--	--	--	0	--	--	--
	**MamGypLTR3a**	LTR	Gypsy	1322	51	3.9%	8.6E-39	1322	56	4.2%	3.4E-38	0	--	--	--	0	--	--	--
	**HUERS-P2-int**	LTR	ERV1	243	28	11.5%	5.6E-34	243	38	15.6%	4.2E-47	0	--	--	--	0	--	--	--
	**LTR1**	LTR	ERV1	242	26	10.7%	1.2E-30	242	36	14.9%	1.0E-43	0	--	--	--	0	--	--	--
	**MER61-int**	LTR	ERV1	1200	40	3.3%	9.4E-28	1200	55	4.6%	2.8E-39	0	--	--	--	0	--	--	--
	**L1P4a**	LINE	L1	572	30	5.2%	3.6E-26	572	23	4.0%	1.9E-14	0	--	--	--	0	--	--	--
	**L1PA17**	LINE	L1	4753	68	1.4%	9.7E-26	4753	62	1.3%	3.8E-15	0	--	--	--	0	--	--	--
	**X1_DNA**	DNA	TcMar-Tigger	670	31	4.6%	1.9E-25	670	29	4.3%	2.0E-19	0	--	--	--	0	--	--	--
	**LTR1A1**	LTR	ERV1	359	24	6.7%	3.7E-23	359	32	8.9%	2.2E-31	0	--	--	--	0	--	--	--
	**THE1B-int**	LTR	ERVL-MaLR	4170	52	1.2%	7.4E-17	4170	57	1.4%	1.1E-14	0	--	--	--	0	--	--	--
	**X12_DNA**	DNA	TcMar-Tigger	1372	30	2.2%	2.0E-15	1372	32	2.3%	9.1E-14	0	--	--	--	0	--	--	--
	**HERVIP10F-int**	LTR	ERV1	302	17	5.6%	9.6E-15	302	27	8.9%	2.6E-26	0	--	--	--	0	--	--	--
	**L1PBa**	LINE	L1	2159	35	1.6%	3.1E-14	2159	37	1.7%	5.9E-12	0	--	--	--	0	--	--	--
	**HERVIP10FH-int**	LTR	ERV1	393	16	4.1%	1.3E-11	393	27	6.9%	3.2E-23	0	--	--	--	0	--	--	--
	**LTR1A2**	LTR	ERV1	804	20	2.5%	7.5E-11	804	30	3.7%	2.5E-18	0	--	--	--	0	--	--	--
	**MER52A**	LTR	ERV1	1382	19	1.4%	5.9E-06	1382	27	2.0%	1.2E-09	0	--	--	--	0	--	--	--
	**MER1A**	DNA	hAT-Charlie	3205	27	0.8%	1.3E-04	3205	52	1.6%	2.6E-16	0	--	--	--	0	--	--	--
	**MER1B**	DNA	hAT-Charlie	5302	33	0.6%	4.4E-03	5302	59	1.1%	2.1E-11	0	--	--	--	0	--	--	--
	**LTR8B**	LTR	ERV1	1601	13	0.8%	0.26	1601	26	1.6%	1.6E-07	0	--	--	--	0	--	--	--
	**LTR16**	LTR	ERVL	3000	19	0.6%	0.30	3000	38	1.3%	2.0E-08	0	--	--	--	0	--	--	--
	**MER41A**	LTR	ERV1	2530	16	0.6%	0.82	2530	30	1.2%	9.7E-06	0	--	--	--	0	--	--	--
	**HERVL-int**	LTR	ERVL	2300	11	0.5%	1	2300	28	1.2%	1.7E-05	0	--	--	--	0	--	--	--
	**L1M3f**	LINE	L1	665	51	7.7%	1.0E-53	665	43	6.5%	1.2E-36	97	4	4.1%	1	97	3	3.1%	1
	**MER20B**	DNA	hAT-Charlie	4099	88	2.1%	4.8E-47	4099	98	2.4%	1.3E-45	818	17	2.1%	1	818	16	2.0%	1
	**Tigger16a**	DNA	TcMar-Tigger	1777	55	3.1%	5.9E-37	1777	61	3.4%	1.6E-36	154	4	2.6%	1	154	4	2.6%	1
	**L1M1**	LINE	L1	9814	115	1.2%	3.0E-36	9814	119	1.2%	2.2E-27	923	2	0.2%	1	923	3	0.3%	1
	**ERV3-16A3_I-int**	LTR	ERVL	6189	91	1.5%	9.7E-36	6189	101	1.6%	5.1E-33	905	7	0.8%	1	905	2	0.2%	1
	**MER66C**	LTR	ERV1	567	34	6.0%	9.0E-32	567	42	7.4%	3.3E-38	18	0	0.0%	1	18	0	0.0%	1
	**HUERS-P3b-int**	LTR	ERV1	519	31	6.0%	8.4E-29	519	40	7.7%	5.2E-37	3	0	0.0%	1	3	0	0.0%	1
	**LTR16C**	LTR	ERVL	6577	82	1.2%	2.8E-27	6577	97	1.5%	2.6E-28	1346	14	1.0%	1	1346	11	0.8%	1
	**AmnSINE1**	SINE	Deu	2603	47	1.8%	1.9E-21	2603	46	1.8%	1.1E-15	1330	17	1.3%	1	1330	15	1.1%	1
	**MER135**	DNA	DNA	818	29	3.5%	1.7E-20	818	25	3.1%	4.6E-13	287	5	1.7%	1	287	5	1.7%	1
	**MER119**	DNA	hAT-Charlie	1202	28	2.3%	5.2E-15	1202	32	2.7%	2.2E-15	401	8	2.0%	1	401	10	2.5%	1
	**MER52-int**	LTR	ERV1	489	19	3.9%	1.1E-13	489	25	5.1%	2.8E-18	204	1	0.5%	1	204	0	0.0%	1
	**LTR33**	LTR	ERVL	9171	71	0.8%	1.7E-12	9171	124	1.4%	6.4E-33	1457	10	0.7%	1	1457	13	0.9%	1
	**MER74A**	LTR	ERVL	1556	28	1.8%	3.2E-12	1556	35	2.2%	1.1E-14	731	6	0.8%	1	731	1	0.1%	1
	**LTR33A_**	LTR	ERVL	1644	27	1.6%	8.2E-11	1644	35	2.1%	5.5E-14	476	5	1.1%	1	476	4	0.8%	1
	**LTR16A2**	LTR	ERVL	1817	24	1.3%	1.5E-07	1817	30	1.7%	4.8E-09	416	4	1.0%	1	416	2	0.5%	1
	**MER102b**	DNA	hAT-Charlie	3116	30	1.0%	1.5E-06	3116	37	1.2%	2.1E-07	499	3	0.6%	1	499	3	0.6%	1
	**MER91A**	DNA	hAT-Tip100	3553	32	0.9%	2.2E-06	3553	42	1.2%	1.6E-08	368	6	1.6%	1	368	2	0.5%	1
	**LTR16A1**	LTR	ERVL	2680	26	1.0%	1.5E-05	2680	36	1.3%	1.3E-08	650	6	0.9%	1	650	0	0.0%	1
	**MER102a**	DNA	hAT-Charlie	2999	25	0.8%	4.4E-04	2999	37	1.2%	7.4E-08	384	2	0.5%	1	384	1	0.3%	1
	**ERVL-B4-int**	LTR	ERVL	3855	29	0.8%	4.6E-04	3855	48	1.2%	1.0E-10	1391	5	0.4%	1	1391	6	0.4%	1

	**LTR33A**	LTR	ERVL	2022	19	0.9%	1.8E-03	2022	28	1.4%	1.1E-06	504	7	1.4%	1	504	0	0.0%	1
	**MER102c**	DNA	hAT-Charlie	3106	22	0.7%	0.02	3106	35	1.1%	2.3E-06	364	4	1.1%	1	364	4	1.1%	1
	**LTR16E1**	LTR	ERVL	2454	18	0.7%	0.08	2454	32	1.3%	3.5E-07	509	5	1.0%	1	509	3	0.6%	1
**Mouse-Specific**	**B2_Mm2**	SINE	B2	0	--	--	--	0	--	--	--	88537	7794	8.8%	< 2E-200	88537	7561	8.5%	< 2E-200
	**B3**	SINE	B2	0	--	--	--	0	--	--	--	144792	8832	6.1%	< 2E-200	144792	8774	6.1%	< 2E-200
	**B3A**	SINE	B2	0	--	--	--	0	--	--	--	91641	4650	5.1%	< 2E-200	91641	4552	5.0%	< 2E-200
	**B2_Mm1t**	SINE	B2	0	--	--	--	0	--	--	--	22963	1720	7.5%	< 2E-200	22963	1859	8.1%	< 2E-200
	**B2_Mm1a**	SINE	B2	0	--	--	--	0	--	--	--	18277	676	3.7%	9.7E-185	18277	798	4.4%	9.6E-286
	**IAPEY4_LTR**	LTR	ERVK	0	--	--	--	0	--	--	--	88	65	73.9%	1.4E-108	88	74	84.1%	1.7E-134
	**ORR1E**	LTR	ERVL-MaLR	0	--	--	--	0	--	--	--	27812	655	2.4%	3.6E-90	27812	532	1.9%	6.6E-56
	**ORR1D2**	LTR	ERVL-MaLR	0	--	--	--	0	--	--	--	15011	393	2.6%	3.6E-65	15011	323	2.2%	3.7E-43
	**RMER1A**	Other	Other	0	--	--	--	0	--	--	--	3788	148	3.9%	1.9E-42	3788	79	2.1%	4.8E-09
	**RMER1B**	Other	Other	0	--	--	--	0	--	--	--	7250	158	2.2%	8.0E-18	7250	90	1.2%	0.63
			**Shared Total**	25148	1342	5.3%	--	25148	1400	5.6%	--	7924	375	4.7%	--	7924	338	4.3%	--
			**Human-Specific Total**	114948	2165	1.9%	--	114948	2634	2.3%	--	102772	142	0.1%	--	102772	108	0.1%	--
			**Mouse-Specific Total**	0	--	--	--	0	--	--	--	420159	25091	6.0%	--	420159	24642	5.9%	--

Inidvidual one-sided binomial tests for enrichment of CTCF binding sites were performed for all TE types in which CTCF-binding was observed within at least 25 copies in at least one of the four cell types used: human K562 and GM12878, and mouse CH12 and MEL. Binomial expected frequencies were set to the observed frequency of CTCF binding within all repeat annotations within each cell type. Significant enrichment of CTCF binding for a given TE type in a given cell type was based on three criteria: a Bonferroni-corrected p-value <= 1x10-4, a minimum of 25 TE copies with CTCF binding, and at least 1% of TE copies occupied by CTCF. TE type, class, and family are give in columns 1-3. Columns 4-7 present the total number of insertions, number of insertions bound by CTCF, percentage of insertions bound by CTCF, and Bonferroni-corrected binomial p-value for each enriched TE type, in number of insertions bound by CTCF, percentage of insertions bound by CTCF, and Bonferroni-corrected binomial p-value for each enriched TE type, in 16-19 for mouse MEL cells. P-values reported as < 2E-200 represent actual values below the precision threshold of the machine on which the analysis was performed.

Supplementary Table 2: CTCF-enriched transposable element types from permutation test procedure

		GM12878							K562							CH12							MEL						
	TE Type	# Bound	% Bound	# Perm Avg	# Perm Min	# Perm Max	% Expected	p-value	# Bound	% Bound	# Perm Avg	# Perm Min	# Perm Max	% Expected	p-value	# Bound	% Bound	# Perm Avg	# Perm Min	# Perm Max	% Expected	p-value	# Bound	% Bound	# Perm Avg	# Perm Min	# Perm Max	% Expected	p-value
Shared	MER20	587	3.5%	2	0	10	0.4%	< 1.9E-300	602	0.4%	2	0	11	3.6%	< 1.9E-300	171	2.9%	0.3	0	4	0.2%	< 1.9E-300	140	2.3%	0.2	0	4	0.2%	< 1.9E-300
	LTR41	134	12.1%	0.04	0	2	0.03%	< 1.9E-300	138	0.02%	0.03	0	3	12.4%	< 1.9E-300	62	12.4%	0.008	0	1	0.01%	1.6E-214	60	12.0%	0.008	0	1	0.01%	4.4E-207
	LTR41B	111	13.3%	0.02	0	2	0.02%	< 1.9E-300	109	0.01%	0.02	0	2	13.1%	< 1.9E-300	47	16.4%	0.004	0	1	0.01%	4.0E-171	50	17.4%	0.004	0	1	0.01%	2.2E-182
	MER91B	113	8.0%	0.04	0	2	0.03%	< 1.9E-300	119	0.03%	0.04	0	2	8.5%	< 1.9E-300	35	15.1%	0.002	0	1	0.01%	1.1E-131	33	14.2%	0.002	0	1	0.01%	1.0E-124
	LTR50	185	7.3%	0.1	0	2	0.1%	< 1.9E-300	199	0.1%	0.1	0	3	7.8%	< 1.9E-300	24	4.4%	0.004	0	1	0.02%	1.5E-79	19	3.5%	0.004	0	1	0.02%	2.8E-61
	L1M1	115	1.2%	0.3	0	3	0.2%	2.9E-254	119	0.2%	0.3	0	6	1.2%	2.9E-266	2	0.2%	0.0003	0	1	0.02%	2.7E-05	3	0.3%	0.001	0	1	0.03%	4.8E-08
	MER20B	88	2.1%	0.08	0	3	0.1%	2.2E-229	98	0.1%	0.09	0	2	2.4%	2.6E-256	17	2.1%	0.004	0	1	0.03%	1.5E-52	16	2.0%	0.004	0	1	0.02%	5.0E-50
	ERV3-16A3_I-int	91	1.5%	0.1	0	4	0.1%	1.1E-219	101	0.1%	0.1	0	4	1.6%	5.8E-245	7	0.8%	0.003	0	1	0.04%	1.2E-19	2	0.2%	0.001	0	1	0.04%	1.8E-04
	LTR16C	82	1.2%	0.1	0	2	0.1%	3.0E-197	97	0.1%	0.1	0	3	1.5%	4.5E-232	14	1.0%	0.004	0	1	0.03%	5.0E-42	11	0.8%	0.004	0	1	0.03%	2.5E-32
	LTR55	53	13.1%	0.005	0	1	0.009%	2.1E-191	50	0.007%	0.004	0	1	12.3%	4.1E-185	10	6.7%	0.0002	0	1	0.002%	1.7E-41	11	7.3%	0.0003	0	1	0.003%	2.5E-44
	MamGypLTR3	55	8.3%	0.007	0	2	0.013%	3.5E-189	53	0.014%	0.007	0	2	8.0%	2.1E-180	17	14.8%	0.001	0	1	0.004%	3.9E-66	17	14.8%	0.0004	0	1	0.002%	2.7E-70
	MLT1I	79	0.7%	0.2	0	3	0.2%	1.3E-172	99	0.2%	0.2	0	3	0.9%	6.5E-216	18	1.0%	0.009	0	2	0.05%	7.5E-51	11	0.6%	0.005	0	1	0.05%	8.4E-31
	L1M3f	51	7.7%	0.009	0	1	0.02%	1.1E-168	43	0.01%	0.006	0	1	6.5%	1.9E-146	0	4.1%	0	0	0	--	1.0E+00	3	3.1%	0.0002	0	1	0.01%	7.5E-10
	Tigger16a	55	3.1%	0.02	0	2	0.04%	7.3E-164	61	0.04%	0.03	0	2	3.4%	3.0E-178	4	2.6%	0.0001	0	1	0.00%	2.5E-15	4	2.6%	0.0004	0	1	0.01%	6.0E-13
	LTR33	71	0.8%	0.1	0	2	0.2%	4.8E-163	124	0.2%	0.2	0	4	1.4%	1.6E-281	10	0.7%	0.004	0	1	0.04%	2.8E-28	13	0.9%	0.005	0	1	0.03%	2.8E-38
	ERVL-E-int	66	0.8%	0.1	0	3	0.2%	1.7E-149	82	0.2%	0.2	0	3	0.9%	4.3E-187	11	1.0%	0.003	0	1	0.03%	1.2E-33	5	0.5%	0.002	0	1	0.05%	3.7E-13
	AmnSINE1	47	1.8%	0.02	0	2	0.1%	2.9E-133	46	0.05%	0.02	0	2	1.8%	2.3E-131	17	1.3%	0.005	0	1	0.03%	3.1E-52	15	1.1%	0.007	0	1	0.05%	1.6E-42
	MER135	29	3.5%	0.004	0	1	0.01%	2.4E-98	25	0.02%	0.005	0	1	3.1%	3.4E-81	5	1.7%	0.001	0	1	0.01%	1.6E-16	5	1.7%	0.001	0	1	0.01%	1.5E-16
	MER119	28	2.3%	0.007	0	1	0.02%	2.1E-88	32	0.03%	0.009	0	1	2.7%	7.5E-99	8	2.0%	0.0003	0	1	0.004%	9.7E-31	10	2.5%	0.001	0	1	0.01%	9.5E-34
	MER91A	32	0.9%	0.03	0	2	0.1%	2.2E-84	42	0.1%	0.03	0	2	1.2%	1.2E-111	6	1.6%	0.001	0	1	0.01%	1.3E-20	2	0.5%	0.0003	0	1	0.02%	2.5E-05
	MER102b	30	1.0%	0.02	0	2	0.1%	1.0E-81	37	0.1%	0.03	0	2	1.2%	4.8E-100	3	0.6%	0.001	0	1	0.02%	1.2E-08	3	0.6%	0.0004	0	1	0.01%	6.0E-09
	MER74A	28	1.8%	0.01	0	2	0.04%	1.2E-80	35	0.03%	0.01	0	1	2.2%	3.0E-106	6	0.8%	0.002	0	1	0.03%	9.5E-18	1	0.1%	0.0003	0	1	0.03%	1.7E-01
	LTR33A_	27	1.6%	0.01	0	2	0.04%	2.5E-79	35	0.04%	0.01	0	2	2.1%	8.0E-103	5	1.1%	0.001	0	1	0.01%	8.4E-16	4	0.8%	0.001	0	1	0.02%	5.6E-12
	ERVL-B4-int	29	0.8%	0.02	0	2	0.1%	9.7E-76	48	0.1%	0.04	0	3	1.2%	7.3E-124	5	0.4%	0.002	0	1	0.04%	2.0E-13	6	0.4%	0.003	0	1	0.04%	1.9E-16
	LTR16A2	24	1.3%	0.01	0	1	0.04%	7.3E-70	30	0.04%	0.01	0	1	1.7%	5.7E-88	4	1.0%	0.001	0	1	0.02%	3.2E-12	2	0.5%	0.0002	0	1	0.01%	1.1E-05
	MER102a	25	0.8%	0.02	0	1	0.1%	8.2E-68	37	0.1%	0.03	0	2	1.2%	3.1E-100	2	0.5%	0.0003	0	1	0.02%	2.7E-05	1	0.3%	0.0001	0	1	0.01%	5.6E-02
	LTR16A1	26	1.0%	0.02	0	1	0.06%	1.9E-71	36	0.06%	0.02	0	2	1.3%	1.9E-98	6	0.9%	0.001	0	1	0.02%	1.5E-18	--	--	--	--	--	--	--
	MER102c	22	0.7%	0.01	0	2	0.1%	4.1E-60	35	0.1%	0.02	0	2	1.1%	1.8E-94	4	1.1%	0.001	0	1	0.02%	3.2E-12	4	1.1%	0.0003	0	1	0.01%	1.9E-13
	LTR16E1	18	0.7%	0.01	0	1	0.1%	4.2E-48	32	0.1%	0.02	0	2	1.3%	9.2E-89	5	1.0%	0.001	0	1	0.02%	1.6E-15	3	0.6%	0.0004	0	1	0.01%	6.0E-09
	MER91C	104	6.4%	0.04	0	2	0.04%	< 1.9E-300	130	0.04%	0.05	0	2	8.0%	< 1.9E-300	9	8.6%	0.0004	0	1	0.004%	4.3E-34	8	7.6%	0.0001	0	1	0.001%	1.4E-34
	MER52-int	19	3.9%	0.002	0	1	0.01%	4.6E-65	25	0.01%	0.003	0	1	5.1%	1.3E-87	1	0.5%	0.0002	0	1	0.02%	1.2E-01	--	--	--	--	--	--	--
	LTR33A	19	0.9%	0.008	0	1	0.04%	7.0E-55	28	0.04%	0.01	0	2	1.4%	3.0E-82	7	1.4%	0.001	0	1	0.01%	9.8E-24	--	--	--	--	--	--	--
Human-Specific	LTR13	203	44.6%	0.03	0	2	0.01%	< 1.9E-300	253	0.01%	0.04	0	3	55.6%	< 1.9E-300	--	--	--	--	--	--	--	--	--	--	--	--	--	--
	L1PA15-16	93	6.8%	0.03	0	2	0.03%	1.6E-284	82	0.03%	0.03	0	2	6.0%	1.6E-251	--	--	--	--	--	--	--	--	--	--	--	--	--	--
	LTR41C	84	11.3%	0.01	0	1	0.02%	1.7E-283	78	0.02%	0.01	0	2	10.5%	2.6E-260	--	--	--	--	--	--	--	--	--	--	--	--	--	--
	HUERS-P3-int	60	9.0%	0.008	0	2	0.0%	2.2E-204	73	0.0%	0.01	0	2	11.0%	3.8E-251	--	--	--	--	--	--	--	--	--	--	--	--	--	--
	L1PA17	68	1.4%	0.08	0	2	0.1%	1.1E-170	62	0.1%	0.07	0	3	1.3%	8.5E-156	--	--	--	--	--	--	--	--	--	--	--	--	--	--
	MamGypLTR3a	51	3.9%	0.02	0	2	0.03%	2.2E-156	56	0.03%	0.02	0	2	4.2%	4.5E-172	--	--	--	--	--	--	--	--	--	--	--	--	--	--
	HERVK-int	41	16.8%	0.003	0	2	0.01%	1.9E-151	44	0.01%	0.003	0	2	18.0%	6.7E-164	--	--	--	--	--	--	--	--	--	--	--	--	--	--
	HERVE-int	37	13.9%	0.003	0	2	0.01%	4.8E-136	30	0.01%	0.002	0	1	11.2%	7.2E-112	--	--	--	--	--	--	--	--	--	--	--	--	--	--
	THE1B-int	52	1.2%	0.05	0	2	0.1%	3.3E-133	57	0.1%	0.06	0	2	1.4%	6.5E-146	--	--	--	--	--	--	--	--	--	--	--	--	--	--
	MER61-int	40	3.3%	0.01	0	1	0.03%	1.3E-122	55	0.03%	0.02	0	2	4.6%	1.8E-170	--	--	--	--	--	--	--	--	--	--	--	--	--	--
	MER66C	34	6.0%	0.004	0	1	0.01%	3.8E-117	42	0.01%	0.006	0	1	7.4%	5.5E-142	--	--	--	--	--	--	--	--	--	--	--	--	--	--
	HUERS-P2-int	28	11.5%	0.002	0	2	0.01%	2.1E-106	38	0.01%	0.003	0	2	15.6%	2.4E-137	--	--	--	--	--	--	--	--	--	--	--	--	--	--
	X1_DNA	31	4.6%	0.005	0	1	0.02%	2.2E-103	29	0.01%	0.004	0	1	4.3%	1.3E-99	--	--	--	--	--	--	--	--	--	--	--	--	--	--
	HUERS-P3b-int	31	6.0%	0.006	0	1	0.02%	2.4E-101	40	0.01%	0.005	0	2	7.7%	3.2E-139	--	--	--	--	--	--	--	--	--	--	--	--	--	--
	L1P4a	30	5.2%	0.004	0	2	0.01%	5.5E-101	23	0.01%	0.003	0	2	4.0%	3.1E-78	--	--	--	--	--	--	--	--	--	--	--	--	--	--
	L1PBa	35	1.6%	0.02	0	2	0.1%	5.9E-99	37	0.05%	0.02	0	1	1.7%	1.0E-106	--	--	--	--	--	--	--	--	--	--	--	--	--	--
	LTR1	26	10.7%	0.002	0	1	0.01%	3.7E-97	36	0.00%	0.001	0	1	14.9%	4.0E-142	--	--	--	--	--	--	--	--	--	--	--	--	--	--
	X12_DNA	30	2.2%	0.009	0	1	0.03%	1.2E-91	32	0.03%	0.01	0	1	2.3%	8.5E-98	--	--	--	--	--	--	--	--	--	--	--	--	--	--
	LTR13_	20	57.1%	0.000	0	1	0.002%	1.1E-86	25	0.002%	0.0004	0	1	71.4%	6.1E-108	--	--	--	--	--	--	--	--	--	--	--	--	--	--
	LTR1A1	24	6.7%	0.003	0	1	0.01%	1.1E-83	32	0.01%	0.003	0	1	8.9%	6.4E-115	--	--	--	--	--	--	--	--	--	--	--	--	--	--
	MER1B	33	0.6%	0.04	0	2	0.1%	1.7E-81	59	0.1%	0.07	0	2	1.1%	2.3E-146	--	--	--	--	--	--	--	--	--	--	--	--	--	--
	MER1A	27	0.8%	0.02	0	2	0.1%	5.4E-75	52	0.1%	0.03	0	2	1.6%	2.6E-142	--	--	--	--	--	--	--	--	--	--	--	--	--	--
	LTR1A2	20	2.5%	0.004	0	1	0.02%	1.4E-63	30	0.01%	0.004	0	1	3.7%	1.7E-102	--	--	--	--	--	--	--	--	--	--	--	--	--	--
	HERVIP10F-int	17	5.6%	0.002	0	2	0.01%	6.2E-60	27	0.01%	0.002	0	3	8.9%	6.0E-100	--	--	--	--	--	--	--	--	--	--	--	--	--	--
	MER52A	19	1.4%	0.005	0	1	0.03%	2.5E-58	27	0.03%	0.008	0	1	2.0%	1.3E-82	--	--	--	--	--	--	--	--	--	--	--	--	--	--
	HERVIP10FH-int	16	4.1%	0.001	0	1	0.01%	2.4E-57	27	0.01%	0.003	0	2	6.9%	3.4E-95	--	--	--	--	--	--	--	--	--	--	--	--	--	--
	LTR16	19	0.6%	0.01	0	1	0.1%	3.7E-52	38	0.1%	0.02	0	2	1.3%	2.7E-106	--	--	--	--	--	--	--	--	--	--	--	--	--	--
	MER41A	16	0.6%	0.009	0	1	0.1%	3.9E-44	30	0.1%	0.02	0	2	1.2%	2.1E-83	--	--	--	--	--	--	--	--	--	--	--	--	--	--
	LTR8B	13	0.8%	0.005	0	1	0.04%	1.1E-37	26	0.03%	0.009	0	1	1.6%	5.5E-78	--	--	--	--	--	--	--	--	--	--	--	--	--	--
	HERVL-int	11	0.5%	0.005	0	1	0.05%	1.8E-30	28	0.1%	0.01	0	2	1.2%	3.3E-79	--	--	--	--	--	--	--	--	--	--	--	--	--	--
Mouse-Specific	B2_Mm1a	--	--	--	--	--	--		--	--	--	--	--	--	--	676	3.7%	4	0	14	0.7%	< 1.9E-300	798	4.4%	5	0	19	0.7%	< 1.9E-300
	B2_Mm1t	--	--	--	--	--	--	--	--	--	--	--	--	--	--	1720	7.5%	13	2	29	0.8%	< 1.9E-300	1859	8.1%	15	2	31	0.8%	< 1.9E-300
	B2_Mm2	--	--	--	--	--	--	--	--	--	--	--	--	--	--	7794	8.8%	226	168	291	2.9%	< 1.9E-300	7561	8.5%	224	165	285	3.0%	< 1.9E-300
	B3	--	--	--	--	--	--	--	--	--	--	--	--	--	--	8832	6.1%	384	314	457	4.4%	< 1.9E-300	8774	6.1%	382	313	467	4.3%	< 1.9E-300
	B3A	--	--	--	--	--	--	--	--	--	--	--	--	--	--	4650	5.1%	123	82	165	2.7%	< 1.9E-300	4552	5.0%	121	79	164	2.7%	< 1.9E-300
	ORR1D2	--	--	--	--	--	--	--	--	--	--	--	--	--	--	393	2.6%	2	0	9	0.4%	< 1.9E-300	323	2.2%	1	0	7	0.4%	< 1.9E-300
	ORR1E	--	--	--	--	--	--	--	--	--	--	--	--	--	--	655	2.4%	5	0	16	0.8%	< 1.9E-300	532	1.9%	4	0	15	0.8%	< 1.9E-300
	RMER1B	--	--	--	--	--	--	--	--	--	--	--	--	--	--	158	2.2%	0.3	0	4	0.2%	< 1.9E-300	90	1.2%	0.2	0	4	0.2%	1.3E-200
	RMER1A	--	--	--	--	--	--	--	--	--	--	--	--	--	--	148	3.9%	0.2	0	3	0.1%	< 1.9E-300	79	2.1%	0.08	0	3	0.1%	1.6E-200
	IAPEY4_LTR	--	--	--	--	--	--	--	--	--	--	--	--	--	--	65	73.9%	0.001	0	1	0.002%	1.7E-276	74	84.1%	0.003	0	1	0.004%	2.5E-293
	RMER1C	--	--	--	--	--	--	--	--	--	--	--	--	--	--	68	1.2%	0.1	0	3	0.2%	9.8E-159	36	0.6%	0.07	0	2	0.2%	4.2E-82
	RLTR6-int	--	--	--	--	--	--	--	--	--	--	--	--	--	--	28	3.0%	0.007	0	2	0.02%	2.6E-88	17	1.8%	0.005	0	1	0.03%	1.7E-51
	RLTR46A2	--	--	--	--	--	--	--	--	--	--	--	--	--	--	19	33.3%	0.0003	0	1	0.00%	5.7E-82	24	42.1%	0.001	0	1	0.00%	4.2E-99
	MuRRS4-int	--	--	--	--	--	--	--	--	--	--	--	--	--	--	25	2.2%	0.008	0	2	0.03%	1.4E-75	22	2.0%	0.007	0	1	0.03%	6.5E-66
	IAPLTR1_Mm	--	--	--	--	--	--	--	--	--	--	--	--	--	--	24	1.6%	0.008	0	1	0.03%	6.0E-72	10	0.7%	0.004	0	1	0.04%	4.2E-28
	RodERV21-int	--	--	--	--	--	--	--	--	--	--	--	--	--	--	25	1.4%	0.01	0	2	0.1%	3.9E-70	17	0.9%	0.007	0	1	0.04%	2.2E-49
	RLTR13D6	--	--	--	--	--	--	--	--	--	--	--	--	--	--	22	2.7%	0.005	0	1	0.02%	8.1E-70	17	2.1%	0.004	0	1	0.02%	2.7E-53
	MERVK26-int	--	--	--	--	--	--	--	--	--	--	--	--	--	--	22	2.2%	0.008	0	1	0.04%	1.7E-65	16	1.6%	0.005	0	1	0.03%	1.5E-48
	RLTR13D3	--	--	--	--	--	--	--	--	--	--	--	--	--	--	18	5.3%	0.002	0	1	0.01%	1.4E-64	9	2.7%	0.001	0	1	0.01%	6.0E-31
	RLTR30	--	--	--	--	--	--	--	--	--	--	--	--	--	--	17	4.4%	0.002	0	1	0.01%	4.9E-60	12	3.1%	0.001	0	1	0.01%	6.6E-41
	MuRRS-int	--	--	--	--	--	--	--	--	--	--	--	--	--	--	16	4.1%	0.002	0	1	0.01%	5.3E-56	10	2.5%	0.001	0	1	0.01%	9.5E-34
	MMERGLN-int	--	--	--	--	--	--	--	--	--	--	--	--	--	--	17	3.1%	0.003	0	1	0.02%	1.2E-55	16	2.9%	0.003	0	1	0.02%	3.2E-51
	RMER17A-int	--	--	--	--	--	--	--	--	--	--	--	--	--	--	16	4.0%	0.002	0	1	0.01%	8.2E-55	19	4.8%	0.002	0	1	0.01%	1.5E-65
	ERVB4_3-I_MM-int	--	--	--	--	--	--	--	--	--	--	--	--	--	--	12	12.4%	0.0001	0	1	0.00%	1.2E-54	15	15.5%	0.0004	0	1	0.00%	4.6E-61
	RLTR30B_MM	--	--	--	--	--	--	--	--	--	--	--	--	--	--	16	5.7%	0.002	0	1	0.01%	1.9E-54	12	4.2%	0.001	0	1	0.01%	2.7E-41
	IAPLTR1a_Mm	--	--	--	--	--	--	--	--	--	--	--	--	--	--	17	0.9%	0.010	0	1	0.1%	1.7E-46	20	1.0%	0.012	0	1	0.1%	4.4E-55
	RLTR46A	--	--	--	--	--	--	--	--	--	--	--	--	--	--	10	14.5%	0.0001	0	1	0.001%	1.6E-44	13	18.8%	0.0003	0	1	0.002%	1.4E-53
	RLTR18-int	--	--	--	--	--	--	--	--	--	--	--	--	--	--	13	2.6%	0.002	0	1	0.01%	4.3E-44	12	2.4%	0.002	0	1	0.01%	3.3E-40
	MMVL30-int	--	--	--	--	--	--	--	--	--	--	--	--	--	--	14	1.6%	0.004	0	1	0.03%	1.8E-42	14	1.6%	0.003	0	1	0.02%	1.2E-43

**RLTR41**	--	--	--	--	--	--	--	--	--	--	--	--	--	--	14	1.3%	0.005	0	1	0.03%	9.5E-42	12	1.1%	0.003	0	1	0.02%	4.1E-37
**ERVB4_1-I_MM-int**	--	--	--	--	--	--	--	--	--	--	--	--	--	--	11	3.8%	0.001	0	2	0.01%	4.3E-38	10	3.5%	0.002	0	1	0.02%	8.9E-33
**MamRep1161**	--	--	--	--	--	--	--	--	--	--	--	--	--	--	10	3.9%	0.001	0	1	0.01%	1.0E-36	7	2.7%	0.001	0	1	0.01%	9.1E-24
**IAPEY4_I-int**	--	--	--	--	--	--	--	--	--	--	--	--	--	--	10	2.2%	0.001	0	1	0.01%	2.3E-33	11	2.4%	0.001	0	1	0.01%	1.0E-37
**IAPLTR2_Mm**	--	--	--	--	--	--	--	--	--	--	--	--	--	--	8	0.4%	0.006	0	1	0.1%	1.2E-20	24	1.1%	0.02	0	2	0.1%	1.9E-64
**RLTR6_Mm**	--	--	--	--	--	--	--	--	--	--	--	--	--	--	3	1.4%	0.0001	0	1	0.003%	1.0E-10	12	5.6%	0.001	0	1	0.01%	3.7E-42
**RLTR46B**	--	--	--	--	--	--	--	--	--	--	--	--	--	--	12	16.7%	0	0	0	--	1.0E+00	10	13.9%	0.0002	0	1	0.002%	1.6E-41

Inidvidual one-sided permutation tests for enrichment of CTCF binding sites were performed for all TE types in which CTCF-binding was observed within at least 25 copies in at least one of the four cell types used: human K562 and GM12878, and mouse CH12 and MEL. Empirical distributions for expected CTCF binding frequency in each TE type were prepared by permuting TE labels within the set of CTCF-bound repeats in each cell type for 10,000 rounds and recording the CTCF binding frequency. One sided p-values were then retrieved by plugging in the observed number of CTCF-bound repeats into the empirical CDF. Significant enrichment of CTCF binding for a given TE type in a given cell type was based on three criteria: a Bonferroni-corrected p-value <= 1x10-4, a minimum of 25 TE copies with CTCF binding, and at least 1% of TE copies occupied by CTCF. For each cell type the number and percentage of TE insertions bound, average, minimum, and maximum number of CTCF-bound copies observed in permutations, expected frequency, and Bonferroni-corrected p-value are given for each enriched TE type in each of the four cell types. P-values reported as < 1.9E-300 represent actual values below the precision threshold of the machine on which the analysis was performed.

Supplementary Table 3: Enrichment of species-specific motif-words in species-specific repeats

		Transposable Elements		Whole-Genome		
		Bound Words	Bound Species-Specific Words	Bound Words	Bound Species-Specific Words	P-Value
Human	LTR13	330	235	923643	27890	3.26E-271
	MER20	693	254	923280	27871	4.12E-193
	L1PB1	226	70	923747	28055	1.31E-46
	THE1B-int	169	105	923804	28020	1.56E-110
	HERVE-int	117	64	923856	28061	8.78E-62
Mouse	B3	9966	3949	505324	95770	< 3.26E-271
	B3A	5236	1792	510054	97927	3.72E-140
	B2_Mm2	20255	16801	495035	82918	< 3.26E-271
	B2_Mm1t	5830	5139	509460	94580	< 3.26E-271
	B2_Mm1a	2167	2038	513123	97681	< 3.26E-271

One-sided Fisher's exact tests were used to test for enrichment of species-specific motif-words within CTCF-bound transposable elements in human and mouse relative to CTCF binding sites not within annotated repeats. The table presents the number of species-specific and non-species-specific words present in each TE type in the human and mouse genomes. A p-value threshold of 1x10-40 was required for significance. P-values reported as <3.26E-271 represent actual p-values below the precision threshold of the machine on which the analysis was performed.

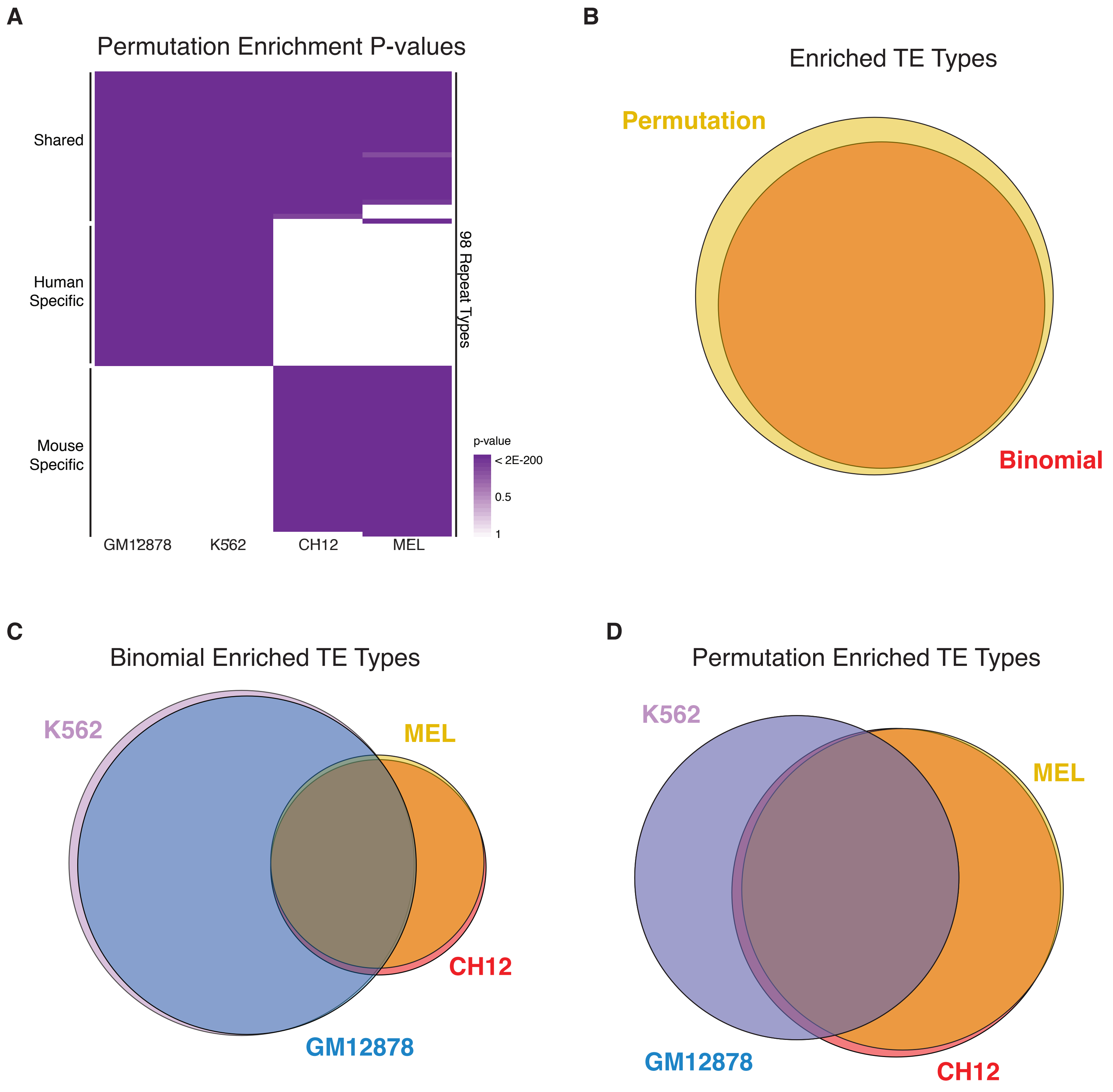

Supplementary Fig. 1: CTCF enrichments are robust to different statistical testing procedures and stable across cell types. A) A permutation-based testing approach was used to retest for CTCF binding enrichments. 98 significant results were recovered. **B)** Permutation tests recovered 100% of TE types found to be enriched in binomial tests. **C-D)** The majority of enriched TE types were detectable across all cell types tested in each species in both binomial and permutation tests.

Supplementary Table 4: Species-specific motif-word occurences in binomial enriched repeats

Group	Repeat	Human: Most Frequent Word	Human: Total Bound Insertions hg19	Human: Bound Word Occurences hg19	Human: Bound Word Occurences mm9	Human: Odds Ratio	Mouse: Most Frequent Word	Mouse: Total Bound Insertions mm9	Mouse: Bound Word Occurences hg19	Mouse: Bound Word Occurences mm9	Mouse: Odds Ratio
Shared	MER20	CTACCCACTAGATGCCAG	693	53	2	4.47	CTATCCACTAGAGGCCAG	174	0	3	-Inf
	LTR41	TTTGTCAGTAGAGGGCGC	149	5	0	Inf	TTGTCCAGTAGATGGCAG	65	0	2	-Inf
	LTR41B	TTTGTCAGTAGAGGGCGC	128	7	0	Inf	ACTGCCAGTAGAGGGGG	43	0	1	-Inf
	MER91B	TTTTCCAGCAGATGGCAG	114	7	1	3.39	ACTCCCAGCAGATGGCA	37	0	1	-Inf
	MamGypLTR3	GCTGCCACATGGTGTCAC	70	2	0	Inf	ACGACCACATGGTGTCAC	15	0	1	-Inf
	LTR50	TCTGCCAATAGGGGGCAC	233	5	0	Inf	ACTGCCTGTAGGGGGCG	26	0	1	-Inf
	MER91C	CACAACTCCAGGGGGCG	91	4	0	Inf	AACAACACCAGGGGGCA	6	0	1	-Inf
	LTR55	ATGACCACGAGGTGGCAG	66	4	0	Inf	ATGACCACAAGGTGGCA	11	1	3	0.09
Human-Specific	LTR13	AGGGCCACCAGAGGGCT	330	152	0	Inf	--	--	--	NA	--
	HERVK-int	CCAGCCACCAGGTTTCAG	79	11	0	Inf	--	--	--	NA	--
	LTR41C	TTTGTCAATAGAGGGTGCT	102	4	0	Inf	--	--	--	NA	--
	L1PA15-16	CAAGCCAGTAGATGGCAC	165	4	0	Inf	--	--	--	NA	--
	HUERS-P3-int	CTGTCCTCTAGGGGGAGC	96	8	0	Inf	--	--	--	NA	--
	HERVE-int	GCTGCCAGCTGAGGACAG	117	17	0	Inf	--	--	--	NA	--
	LTR13_	AGGGCCACTAGAGGGCTC	51	11	0	Inf	--	--	--	NA	--
	MamGypLTR3a	GCTGCCACACGGTGTCGC	63	3	0	Inf	--	--	--	NA	--
	HUERS-P2-int	TCAGCCAGCAGGGGTCGC	69	5	0	Inf	--	--	--	NA	--
	LTR1	CAAACCTCTAGGGGGAGC	66	14	0	Inf	--	--	--	NA	--
	MER61-int	CCTGCTGACAGGGGGCG	107	15	0	Inf	--	--	--	NA	--
	L1P4a	AGAGCCAGCAGGTAGAAA	40	1	0	Inf	--	--	--	NA	--
	L1PA17	CAGTCCAGTAGGTGGCAC	87	3	0	Inf	--	--	--	NA	--
	X1_DNA	ATTGCCACATGGTGGCAC	44	2	0	Inf	--	--	--	NA	--
	LTR1A1	CAAACCTCTAGGGGGAGC	50	4	0	Inf	--	--	--	NA	--
	THE1B-int	AGCTCCACTAGGTGGTGC	169	40	0	Inf	--	--	--	NA	--
	X12_DNA	AACTCCACCAGAGGGTGC	30	1	0	Inf	--	--	--	NA	--
	HERVIP10F-int	GCCTCCAGCAGGTGGGG	51	3	0	Inf	--	--	--	NA	--
	L1PBa	CCAGCCAGGAGGTGGCG	83	8	0	Inf	--	--	--	NA	--
	HERVIP10FH-int	GCCGCCACAAGGGGGCG	47	5	0	Inf	--	--	--	NA	--
	LTR1A2	CAAACCTCTAGGGGGAGC	53	6	0	Inf	--	--	--	NA	--
	MER52A	CTGGCCTGCAGGTGCCCC	83	3	0	Inf	--	--	--	NA	--
	MER1A	TCTGACAGGAGGTGGAGC	80	7	0	Inf	--	--	--	NA	--
	MER1B	TCTGACAGGAGGTGGAGC	57	9	0	Inf	--	--	--	NA	--
	LTR8B	TCAGCCTCTGGGTGGGGC	21	2	0	Inf	--	--	--	NA	--
	LTR16	ACAACCACGGGGAGGCA	27	1	0	Inf	--	--	--	NA	--
	MER41A	AGGACCACCAGAGGTCAC	33	9	0	Inf	--	--	--	NA	--
	HERVL-int	CCTGCCACCAGGTAGCTG	47	4	0	Inf	--	--	--	NA	--
	L1M3f	TTTGGCACTAGATGGCGC	57	3	0	Inf	TTCAGCACTAGATGGCAG	3	0	2	-Inf
	MER20B	ATTCCCCCTAGGGGGCAG	81	2	0	Inf	AAGGCCTCTAGGGGGCA	13	0	1	-Inf
	Tigger16a	ACAACCACTAGGTGCCTC	70	1	0	Inf	ACAACCACCAGGTGGGG	7	0	1	-Inf
	L1M1	ACAGTCAGCAGGTGGCAA	197	3	0	Inf	--	--	--	NA	--
	ERV3-16A3_I-int	TTGGCCAGAAGGGGGAGT	169	2	0	Inf	CCCGTCAAGAGATGGCG	5	0	1	-Inf
	MER66C	GGAACCAGCAGATGGCGA	45	4	0	Inf	--	--	--	NA	--
	HUERS-P3b-int	CTTTCCTCTAGAGGGAGC	42	3	0	Inf	--	--	--	NA	--
	LTR16C	AACACCACCAGGGGGCA	91	1	0	Inf	ACAGCCACTAGAGGGTG	15	0	1	-Inf
	AmnSINE1	ACGAGCAGCAGAGGGCT	27	1	0	Inf	ACAAGCAGCAGGGGGCT	13	0	1	-Inf
	MER135	CAAGCCACCAGGGTGCA	5	1	0	Inf	ATGGTCACTAGGGGGCC	2	0	1	-Inf
	MER119	GTGTCCAACAGATGTCAC	24	2	0	Inf	ACGCCCAACAGGTGTCG	7	0	1	-Inf
	MER52-int	GTCTCCACTAGGGGGAGA	66	3	0	Inf	--	--	--	NA	--
	LTR33	TTGGCCAGTAGGAGGCAC	123	2	0	Inf	ACAGCCTGAAGGGGACA	15	0	1	-Inf
	MER74A	ACCGCCAGCAGGGGTGA	31	1	0	Inf	ACTGCCAGTAGAAGCCAG	3	0	1	-Inf
	LTR33A_	AGGGCCTGAGGGAGGCA	49	2	0	Inf	AGGGCCAGCAGGAGGCG	3	0	1	-Inf
	LTR16A2	AACGCCAGCAGAGTGTGG	30	1	0	Inf	ATGTCCCATAGAGGGCAG	3	0	1	-Inf
	MER102b	ACAGACACCAGATGGTGG	47	1	0	Inf	TAGGCCACAAGGGGTCA	1	0	1	-Inf
	MER91A	ACAGCCAGCAGAGGGGG	37	1	0	Inf	CCCACCACTAGGGGAAC	6	0	1	-Inf
	LTR16A1	ACCTCCACTAGGGGGCA	45	1	0	Inf	GCTGCCTCAAGGTGGAG	2	0	1	-Inf
	MER102a	AAGGCCTGTAGGGGTCAG	29	1	0	Inf	CCTGACTGCAGGGGGCA	1	0	1	-Inf
	ERVL-B4-int	GTGCCCAATAGATGGTGC	52	2	0	Inf	ATGGCCACATGGTGGAGC	4	0	1	-Inf
	LTR33A	ACTACCCTCAGGGGGAAG	32	1	0	Inf	ACTGCCACCAGGTAGCA	10	0	1	-Inf
	MER102c	AATTCCTCTAGGGGGCAG	25	1	0	Inf	CCAGCCAGCAGGGGGAG	7	0	1	-Inf
	LTR16E1	ACAGCCTAGAGGTGCAGG	21	1	0	Inf	GTGGACAGCAGGGGGCG	2	0	1	-Inf
Mouse-Specific	B2_Mm2	--	--	NA	--	--	TGAGCCACCATGTGGTTG	20255	0	3536	-Inf
	B3	--	--	NA	--	--	GAGGCCAGAAGAGGGCA	9966	0	546	-Inf
	B3A	--	--	NA	--	--	GAGGCCAGAAGAGGGCA	5236	0	202	-Inf
	B2_Mm1t	--	--	NA	--	--	TGAGCCACCATGTGGTTG	5830	0	1376	-Inf
	B2_Mm1a	--	--	NA	--	--	TGAGCCACCATGTGGTTG	2167	0	776	-Inf
	IAPEY4_LTR	--	--	NA	--	--	GCCGCCACTAGATGGTGC	73	0	49	-Inf
	ORR1E	--	--	NA	--	--	GTCCCCAGCTGGTGGCA	929	0	9	-Inf
	ORR1D2	--	--	NA	--	--	GTCCCCAGTTGGTGGCAC	510	0	9	-Inf
	RMER1A	--	--	NA	--	--	TGCACCACCAGGGTGAG	282	0	8	-Inf
	RMER1B	--	--	NA	--	--	CACACCACCAGGGTGAG	226	0	5	-Inf

The most-frequent motif-word in each CTCF-enriched TE was identified in each species. Species-specific motif-words are presented in red. Odds ratios for species-specific occupancy within each TE type, calculated according to Equations 1-2, are given in the final column for each species.

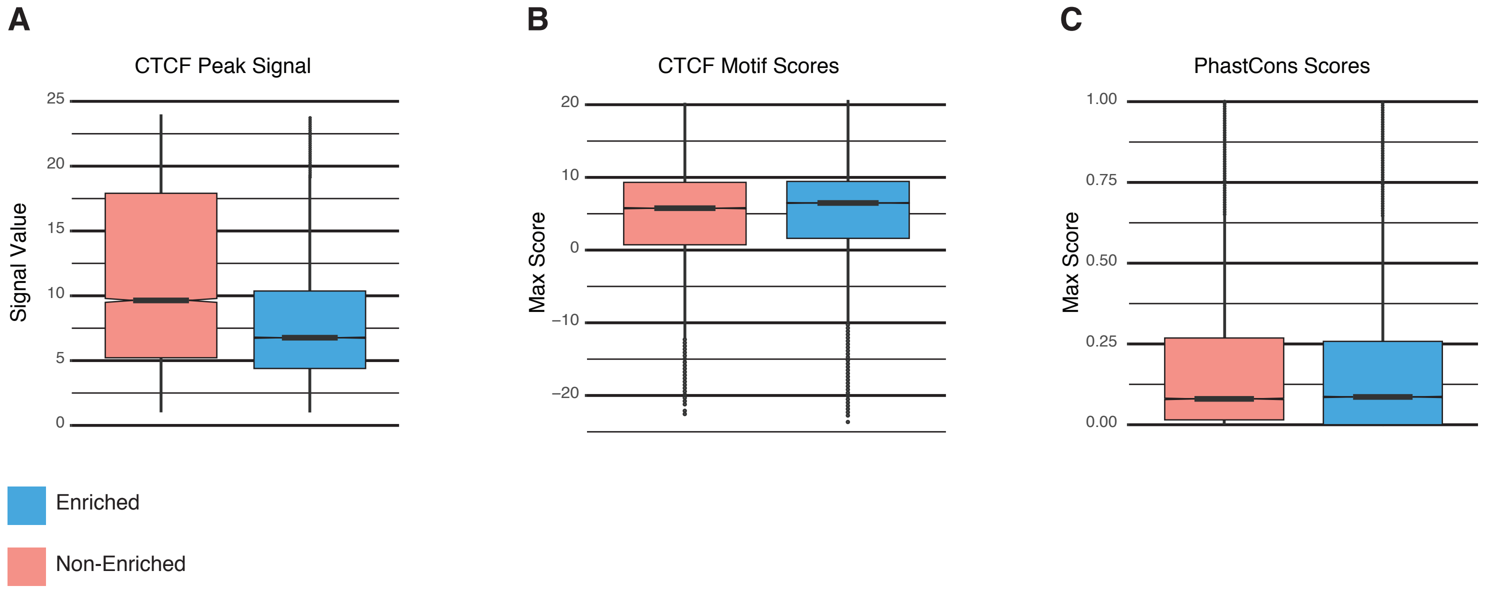

Supplementary Fig. 2: Differences in functional annotation associations do not appear to explain CTCF-binding enrichments in human and mouse TE types. Box plots illustrate score distributions for three functional annotations associated with instances of CTCF-enriched and non-enriched TE types. We wanted to see if enriched TE types displayed any systematic differences in functional properties that might explain their enrichment for CTCF binding. Boxplots are centered around the median, with upper and lower hinges indicating the first and third quartiles. Upper and lower whiskers extend from the hinge to the largest and smallest values within 1.5 × the inter-quartile range from the hinge. Individual data points beyond the ends of the whiskers represent outliers. We considered three different functional annotations that might explain differential CTCF enrichment: **A)** CTCF ChIP-seq peak signal intensity. If CTCF binds more strongly to sites within enriched TE types, it might explain why they appear in enrichment tests. Surprisingly, these scores are actually systematically lower in CTCF-enriched TE instances compared to non-enriched TE types. **B)** We see no systematic differences in CTCF motif scores within enriched and non-enriched TE instances. This suggests that extant copies of both classes of TEs may be equally capable of binding CTCF, and fails to explain differential enrichments. **C)** PhastCons conservation scores show no signficant differences in purifying selection between CTCF sites within enriched and non-enriched TEs, suggesting that both classes are equally likely to experience functional constraint.

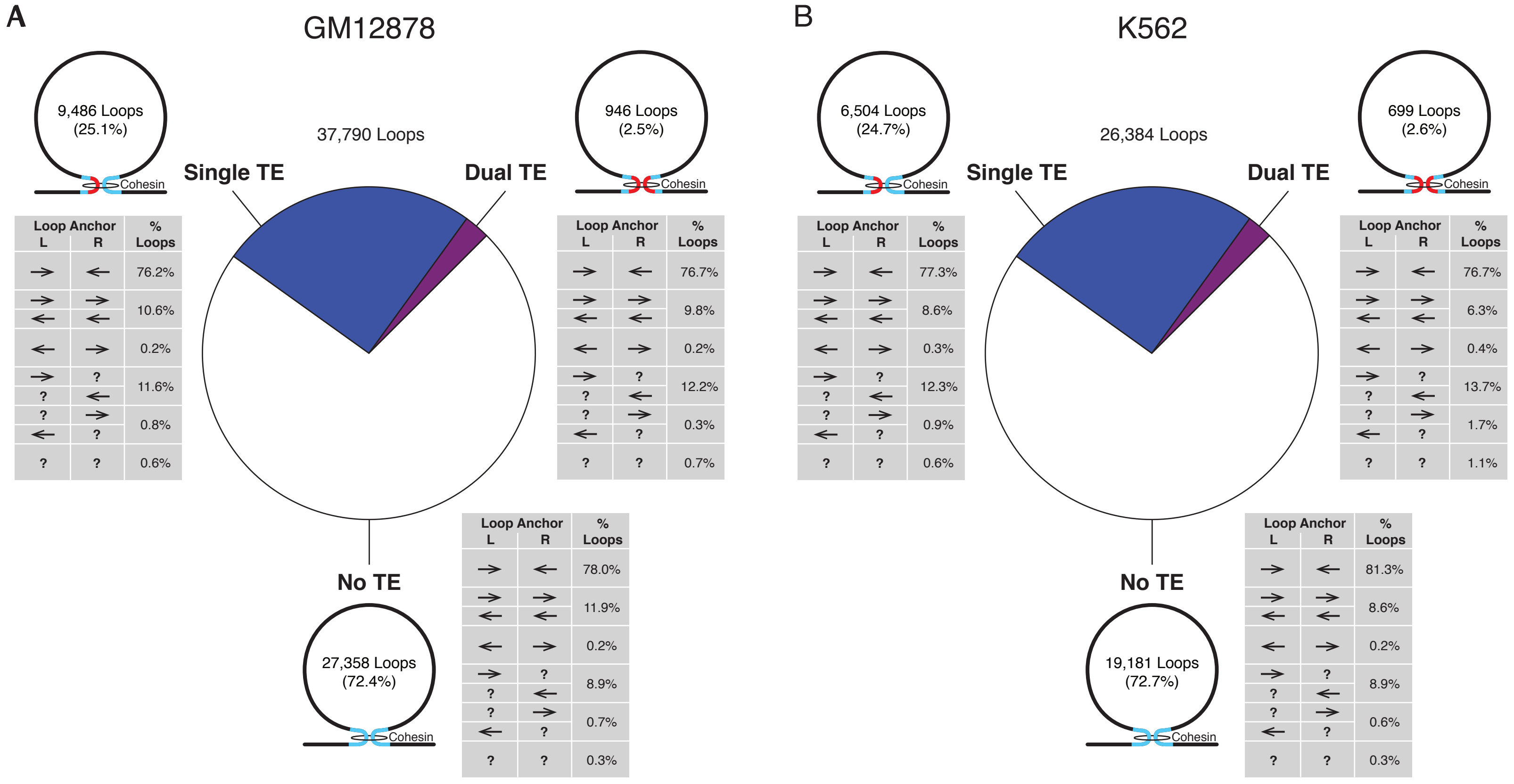

GM12878 – Single TE

Loop Anchor L	R	% Loops
→	←	76.2%
→ / ←	→ / ←	10.6%
←	→	0.2%
→ / ?	? / ←	11.6%
? / ←	→ / ?	0.8%
?	?	0.6%

GM12878 – Dual TE

Loop Anchor L	R	% Loops
→	←	76.7%
→ / ←	→ / ←	9.8%
←	→	0.2%
→ / ?	? / ←	12.2%
? / ←	→ / ?	0.3%
?	?	0.7%

GM12878 – No TE

Loop Anchor L	R	% Loops
→	←	78.0%
→ / ←	→ / ←	11.9%
←	→	0.2%
→ / ?	? / ←	8.9%
? / ←	→ / ?	0.7%
?	?	0.3%

K562 – Single TE

Loop Anchor L	R	% Loops
→	←	77.3%
→ / ←	→ / ←	8.6%
←	→	0.3%
→ / ?	? / ←	12.3%
? / ←	→ / ?	0.9%
?	?	0.6%

K562 – Dual TE

Loop Anchor L	R	% Loops
→	←	76.7%
→ / ←	→ / ←	6.3%
←	→	0.4%
→ / ?	? / ←	13.7%
? / ←	→ / ?	1.7%
?	?	1.1%

K562 – No TE

Loop Anchor L	R	% Loops
→	←	81.3%
→ / ←	→ / ←	8.6%
←	→	0.2%
→ / ?	? / ←	8.9%
? / ←	→ / ?	0.6%
?	?	0.3%

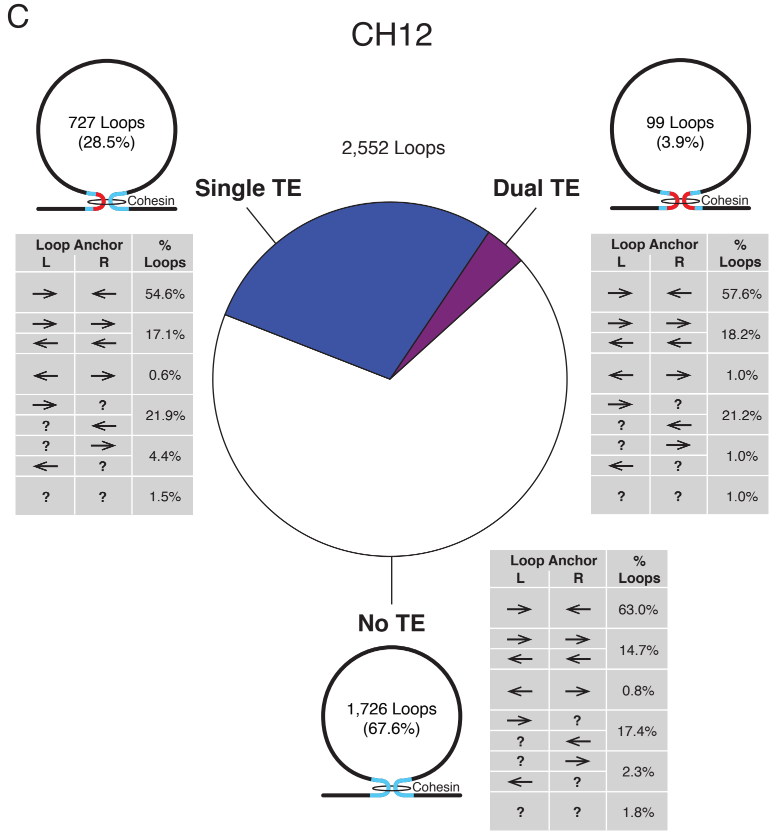

CH12 – Single TE

Loop Anchor L	R	% Loops
→	←	54.6%
→ / ←	→ / ←	17.1%
←	→	0.6%
→ / ?	? / ←	21.9%
? / ←	→ / ?	4.4%
?	?	1.5%

CH12 – Dual TE

Loop Anchor L	R	% Loops
→	←	57.6%
→ / ←	→ / ←	18.2%
←	→	1.0%
→ / ?	? / ←	21.2%
? / ←	→ / ?	1.0%
?	?	1.0%

CH12 – No TE

Loop Anchor L	R	% Loops
→	←	63.0%
→ / ←	→ / ←	14.7%
←	→	0.8%
→ / ?	? / ←	17.4%
? / ←	→ / ?	2.3%
?	?	1.8%

Supplementary Fig. 3: TE-loop associations are stable across species and cell lines. Fractions of TE-derived human RAD21-mediated chromatin loops and mouse Hi-C loops across three cell lines. Pie charts illustrate proportions of loops constructed using one, two, or no TE-derived loop anchors. Associated numbers for each of these three classes are given within the corresponding loop diagrams, and the fraction of those loops displaying every possible CTCF motif layout are given in the associated tables. Observations show very little variation across all three cell types: **A)** Human GM12878 cells **B)** Human K562 cells **C)** Mouse CH12 cells.

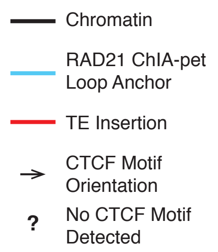

Supplementary Table 5: CTCF motif orientations in TE-derived and native chromatin loops

	Motif Arrangement	Single TE Count	Single TE %	Dual TE Count	Dual TE %	No TE Count	No TE %	All Count	All %
GM12878 RAD21 ChIA-pet	Total Loops	9486	25.1%	946	2.5%	27358	72.4%	37790	100.0%
	Convergent Motifs	7230	76.2%	726	76.7%	21335	78.0%	29291	77.5%
	Tandem Motifs	1005	10.6%	93	9.8%	3255	11.9%	4353	11.5%
	Divergent Motifs	16	0.2%	2	0.2%	68	0.2%	86	0.2%
	Single Motif, Inward-Pointing	1100	11.6%	115	12.2%	2434	8.9%	3649	9.7%
	Single Motif, Outward-Pointin	75	0.8%	3	0.3%	194	0.7%	272	0.7%
	No Motif Detected	60	0.6%	7	0.7%	72	0.3%	139	0.4%
K562 RAD21 ChIA-pet	Total Loops	6504	24.7%	699	2.6%	19181	72.7%	26384	100.0%
	Convergent Motifs	5026	77.3%	536	76.7%	15600	81.3%	21162	80.2%
	Tandem Motifs	560	8.6%	44	6.3%	1659	8.6%	2263	8.6%
	Divergent Motifs	20	0.3%	3	0.4%	41	0.2%	64	0.2%
	Single Motif, Inward-Pointing	800	12.3%	96	13.7%	1711	8.9%	2607	9.9%
	Single Motif, Outward-Pointin	61	0.9%	12	1.7%	108	0.6%	181	0.7%
	No Motif Detected	37	0.6%	8	1.1%	62	0.3%	107	0.4%
CH12 Hi-C	Total Loops	727	28.5%	99	3.9%	1726	67.6%	2552	100.0%
	Convergent Motifs	397	54.6%	57	57.6%	1088	63.0%	1542	60.4%
	Tandem Motifs	124	17.1%	18	18.2%	254	14.7%	396	15.5%
	Divergent Motifs	4	0.6%	1	1.0%	13	0.8%	18	0.7%
	Single Motif, Inward-Pointing	159	21.9%	21	21.2%	300	17.4%	480	18.8%
	Single Motif, Outward-Pointin	32	4.4%	1	1.0%	40	2.3%	73	2.9%
	No Motif Detected	11	1.5%	1	1.0%	31	1.8%	43	1.7%

The orientations of the strongest CTCF binding motifs within both anchors of chromatin loops containing 0, 1, or 2 TE-derived anchors. Convergent orientation indicates a CTCF motif on the + strand within the upstream loop anchor paired with a - strand CTCF motif within the downstream anchor. Tandem motifs are either +/+ or -/- in the upstreamand downstream anchors. Divergent are -/+ in upstream and downstream anchors, respectively. Single motif, inward pointing indicates either a + strand motif in the upstream anchor or a - strand motif in the downstream anchor and no annotated motif in the paired anchor. Single motif, outward pointing posess either a - strand motif in the upstream anchor or a + strand motif in the downstream anchor, with no motif identified in the paired anchor.

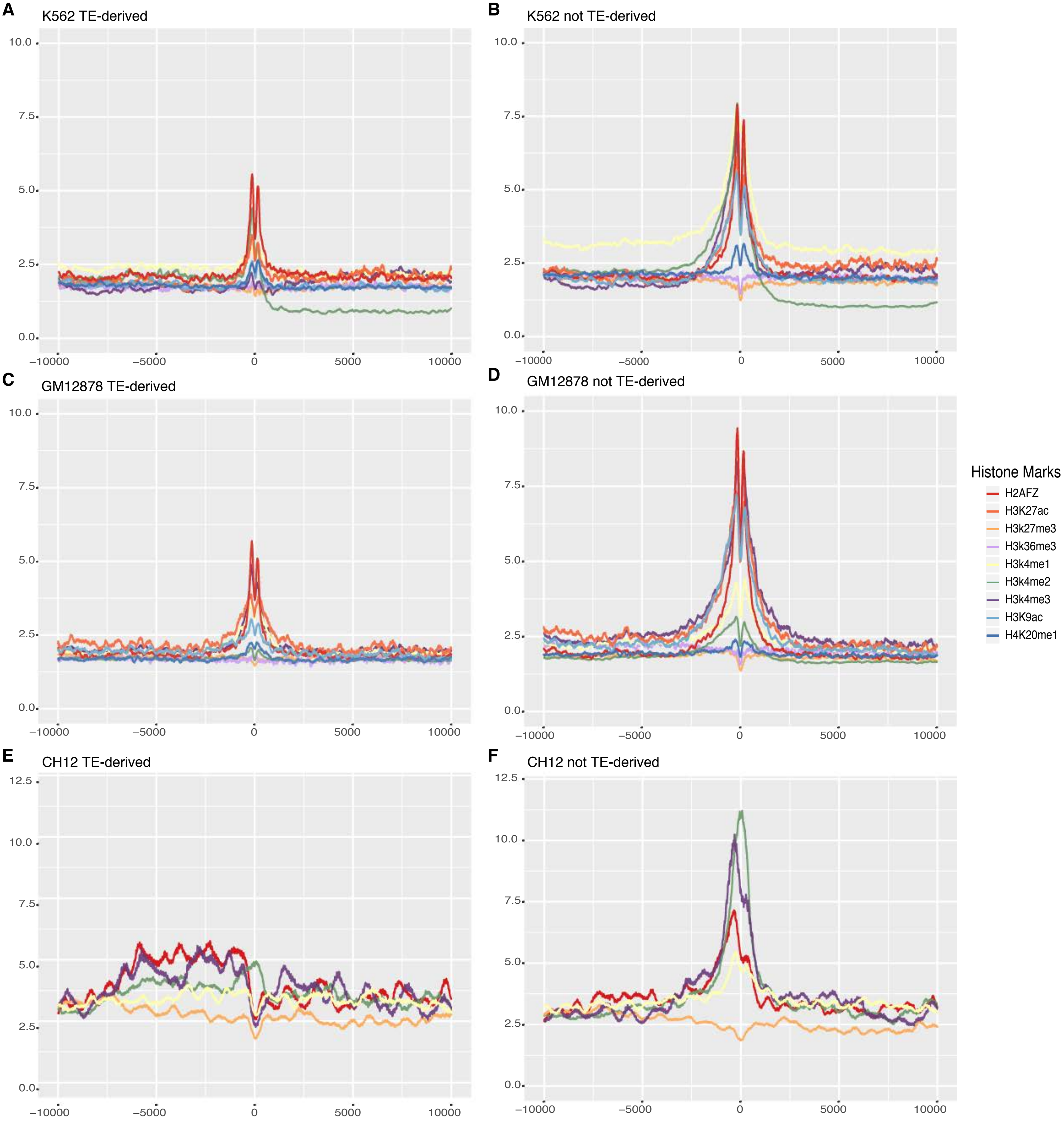

Supplementary Fig. 4: TE-derived vs not TE-derived loop anchors comparison. Average histone modification signals are centered at CTCF peak summits in loop anchors that are (a, c, e) TE-derived or (b, d, f) not TE-derived in K562, GM12878, and CH12, normalized by signals at randomly chosen sites in corresponding cell types. e) TE-derived CH12 loops. Although histone mark enrichments around TE-derived loop anchors in CH12 are of similar magnitude to those in the human cell types, they do not form a distinct peak. We believe this is likely an artifact of decreased read mappability within mouse repetitive sequences.

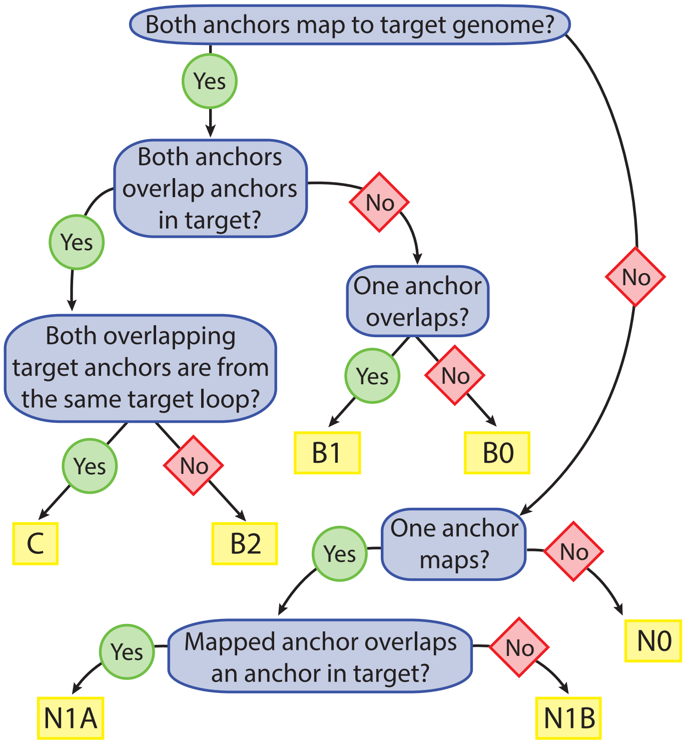

Supplementary Fig. 5: Conservation class assignment algorithm. The process of assigning loops to conservation classes can be described as a decision tree, in which anchor mappings between query and target genomes are considered first, after which query anchors that do map to the target genome are checked for overlap(s) with loop anchors detected in the target dataset.

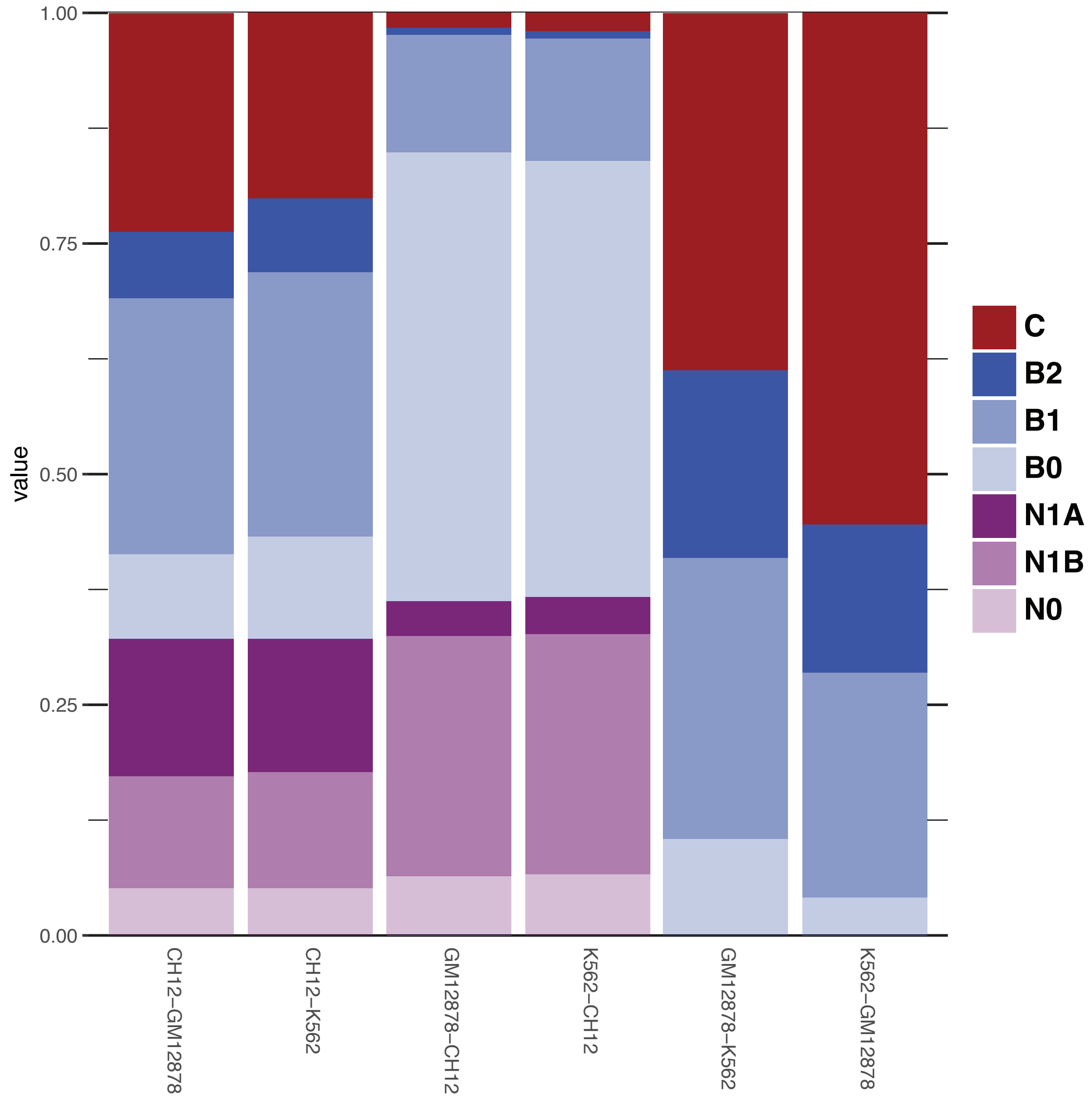

Supplementary Fig. 6: Loop conservation levels are stable in cross-cell and cross-species comparisons. Conservation class breakdowns are shown for all pairwise combinations of cell types.

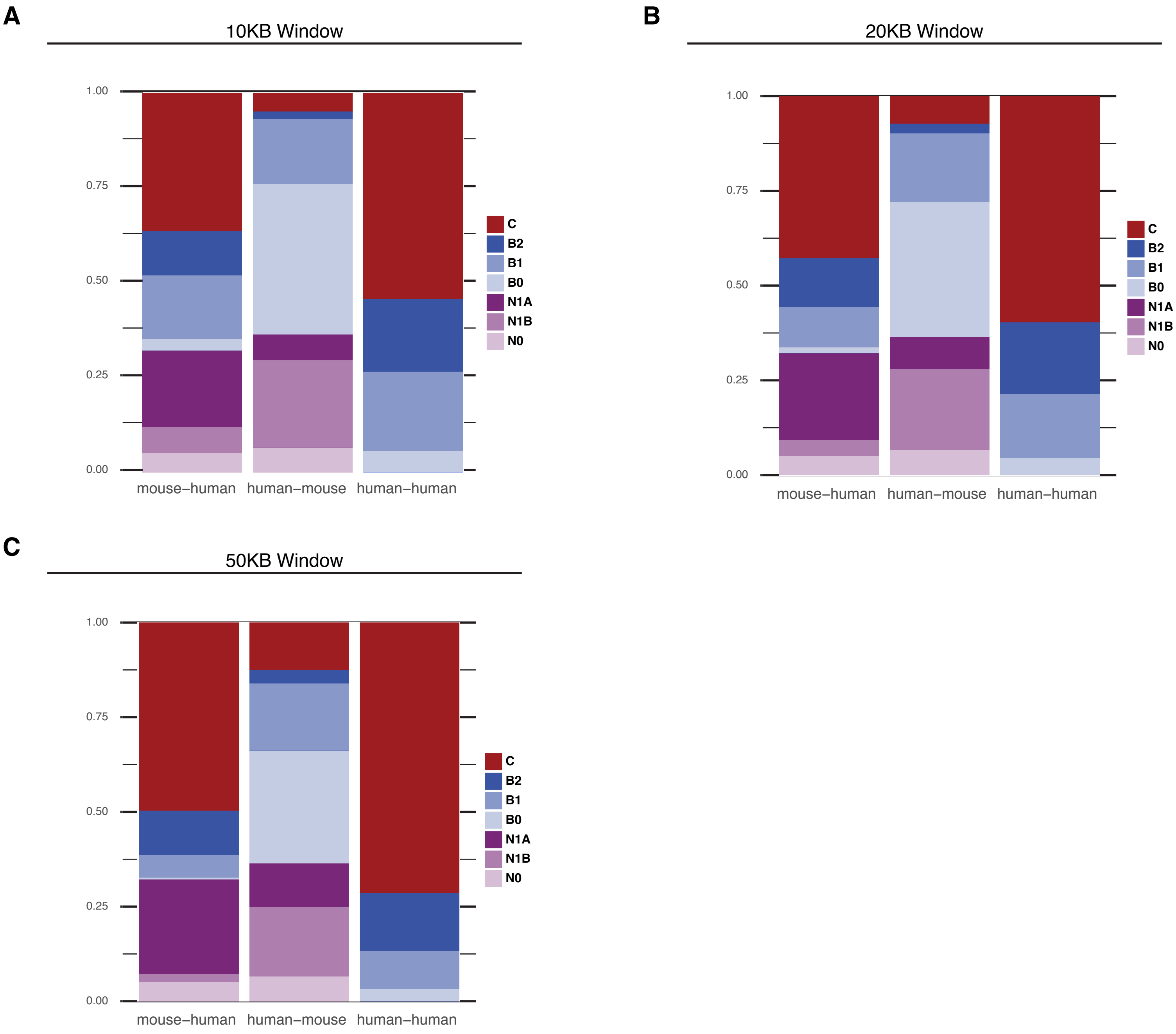

Supplementary Fig. 7:Loop comparison resolution has minmal effects on conservation class membership. The stringency for loops to be considered conserved was varied by extending anchor loci to windows varying from 10-50kb surrounding the center of the ChIP-seq peak annotation. **A)** Conservation class breakdown in each conservation class with 10kb resolution. **B)** Conservation class breakdown in each conservation class with 20kb resolution. **C)** Conservation class breakdown in each conservation class with 50kb resolution.

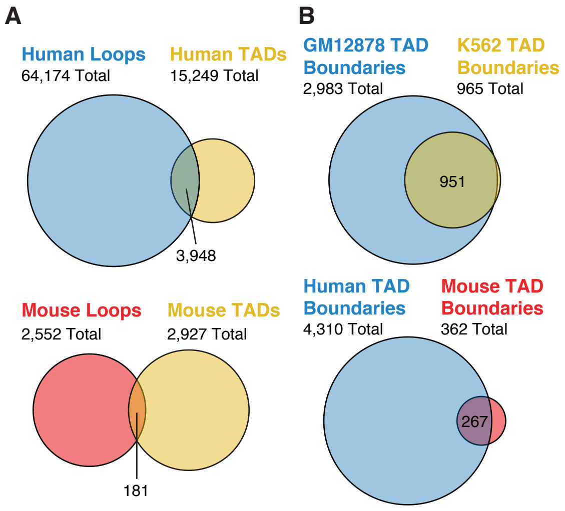

Supplementary Fig. 8: Overlaps between loops in the present dataset and known TADs and TAD boundaries. We aligned chromatin loops with previously annotated TADs {Rao:2014eo}, requiring that both loop anchors map to a coherent set of TAD boundaries to call a match. **(A)** Our dataset captured 26% of human TADs and 6% of mouse TADs in the same cell types when strict anchor matching was enforced. **(B)** Upon closer examination, we noted that conservation metrics reported in previous studies were based only on overlap between individual TAD boundaries, not the entire interval spanned by the TADs being compared {Dixon:2012fb}{VietriRudan:2015gl}. When we compared TAD boundary overlap in an analogous manner, our dataset captured 48% of mouse TADs and 55% of human TADs, and the interspecies and intercell overlaps we observed were consistent with previous reports {Dixon:2012fb}{VietriRudan:2015gl}.

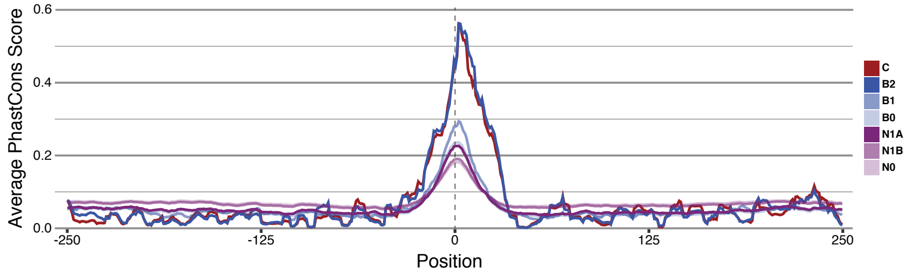

Supplementary Fig. 9: All conservation classes of TE-derived loops show evidence of natural selection. PhastCons scores in a 500bp window surrounding the CTCF peak summit embedded in TE-derived loop anchors are plotted. The strength of conservation at the CTCF binding site appears to be correlated with the degree of loop conservation, following the same pattern observed for non-TE loops.

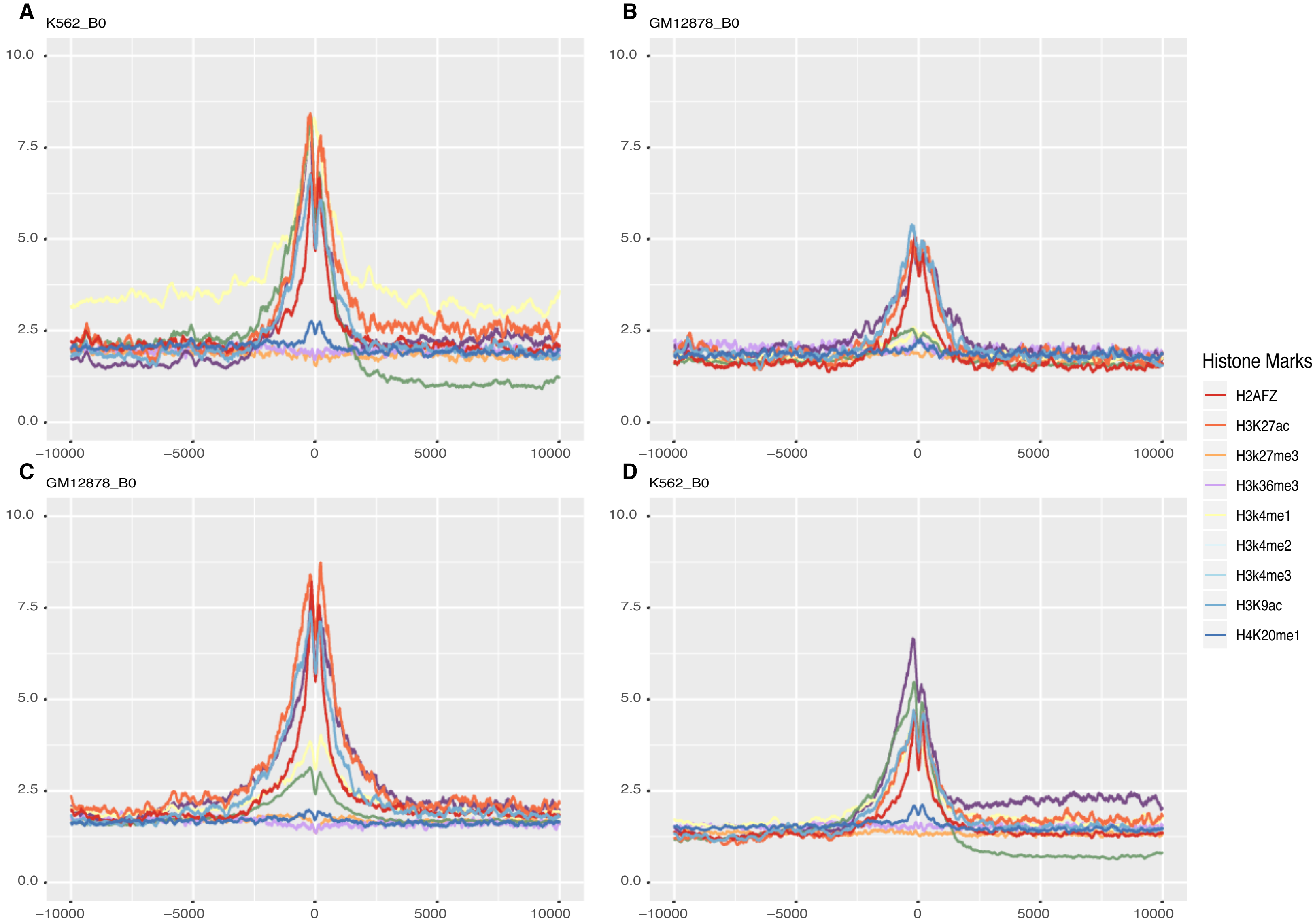

Supplementary Fig. 10: Comparison of histone modifications for non-conserved loops. Average histone modification signals are centered at CTCF peak summits in non-conserved loop anchors of the B0 class that are TE-derived in cross-cesl comparisons between human K562 and GM12878 cells. Signals are normalized against randomly chosen sites in corresponding cell types. A) Histone modification scores for K562 loops not found in GM12878. B) Histone modification scores for GM12878 at loci for loop anchors depicted in (A). C) Histone modification scores for GM12878 loops not found in GM12878. D) Histone modification scores for K562 at loci for loop anchors depicted in (C).

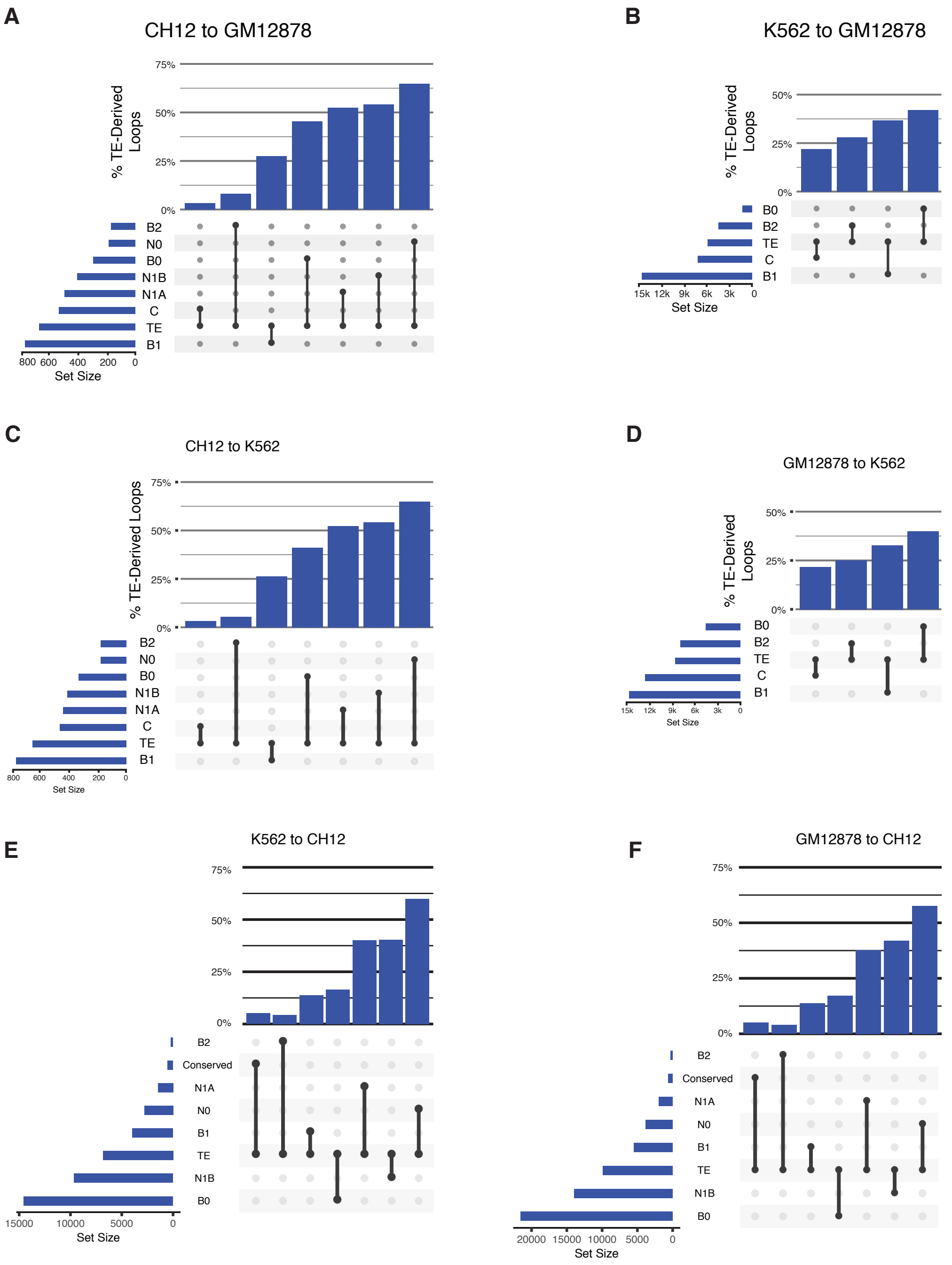

Supplementary Fig. 11: Associations between transposable elements and conservation classes are stable across cell lines. The fraction of loops in each conservation class derived from TE insertions is shown for pairwise comparisons between mouse and human cells, and between different human cell types. All comparisons show a trend toward greater TE-derived contributions as conservation decreases. **A)** Mouse CH12 cells compared to human GM12878 cells. **B)** Human K562 cells compared to human GM12878 cells. **C)** Mouse CH12 cells compared to human K562 cells. **D)** Human GM12878 cells compared to human K562 cells. **E)** Human K562 cells compared to Mouse CH12 cells. **F)** Human GM12878 cells compared to Mouse CH12 cells.

Supplementary Table 6: Membership in loop conservation classes.

Cell		Category															
Query	Target	C				B2				B1				B0			
		All Loops		TE-Derived Loops		All Loops		TE-Derived Loops		All Loops		TE-Derived Loops		All Loops		TE-Derived Loops	
CH12	GM12878	605	23.7%	20	3.3%	184	7.2%	15	8.2%	708	27.7%	194	27.4%	235	9.2%	107	45.5%
	K562	513	20.1%	17	3.3%	204	8.0%	11	5.4%	732	28.7%	192	26.2%	283	11.1%	116	41.0%
GM12878	CH12	614	1.6%	31	5.0%	282	0.7%	11	3.9%	4820	12.8%	664	13.8%	18391	48.7%	3135	17.0%
	K562	14630	38.7%	3166	21.6%	7694	20.4%	1920	25.0%	11510	30.5%	3773	32.8%	3956	10.5%	1573	39.8%
K562	CH12	522	2.0%	27	5.2%	210	0.8%	9	4.3%	3493	13.2%	485	13.9%	12490	47.3%	2065	16.5%
	GM12878	14630	55.5%	3208	21.9%	4237	16.1%	1186	28.0%	6432	24.4%	2353	36.6%	1085	4.1%	456	42.0%

Cell		Category														
Query	Target	N1A				N1B				N0				Total		
		All Loops		TE-Derived Loops		All Loops		TE-Derived Loops		All Loops		TE-Derived Loops		All Loops	TE-Derived Loops	
CH12	GM12878	380	14.9%	199	52.4%	309	12.1%	167	54.0%	131	5.1%	85	64.9%	2552	787	30.8%
	K562	368	14.4%	192	52.2%	321	12.6%	174	54.2%	131	5.1%	85	64.9%		787	30.8%
GM12878	CH12	1417	3.7%	535	37.8%	9825	26.0%	4127	42.0%	2441	6.5%	1408	57.7%	37790	9911	26.2%
	K562	--	--	--	--	--	--	--	--	--	--	--	--		10432	27.6%
K562	CH12	1051	4.0%	422	40.2%	6866	26.0%	2772	40.4%	1752	6.6%	1049	59.9%	26384	6829	25.9%
	GM12878	--	--	--	--	--	--	--	--	--	--	--	--		7203	27.3%

Cell		Species/Cell-Specific TE Content					
Query	Target	All		Mappable		Unmappable	
CH12	GM12878	798	45.3%	301	31.9%	451	55.0%
	K562	805	43.9%	308	30.3%	451	55.0%
GM12878	CH12	10902	29.5%	3799	16.4%	6070	44.4%
	K562	5346	34.6%	5346	34.6%	--	--
K562	CH12	7496	29.2%	2550	16.0%	4243	43.9%
	GM12878	2809	37.4%	2809	37.4%	--	--

The number of total and TE-derived loops for each conservation class is given for all pairwise comparisons of cells from human and mouse. Conservation classes describe decreasing degrees of conservation across cells and/or species, based on the presence of an orthologous sequence in the target genome (mappability), and whether the orthologous sequence is used as a loop anchor in the target species. C = Conserved: Both anchor sequences map and overlap anchors in the same target loop. B2 = Partially conserved: Both anchors map but left and right anchors map to target anchors are from different loops. B1 = Partially conserved: Both anchors map, but only one query anchor overlaps a target loop anchor. B0 = Not conserved: Both anchors map, but neither query anchor overlaps a target loop anchor. N1A = Partially conserved: Only one query anchor maps. The mappable query anchor overlaps a target loop anchor. N1B = Not conserved: Only one query anchor maps. The mappable anchor does not overlap a target loop anchor. N0 = Not conserved: Neither query anchor maps. The “Species/Cell-Specific TE Content” section describes the total contribution of TE-derived loop anchors to all nonconserved loops (classes B1, B0, N1A, N1B, and N0), mappable nonconserved loops (B1 and B0), and unmappable nonconserved loops (N1A, N1B, N0) in each pairwise comparison.

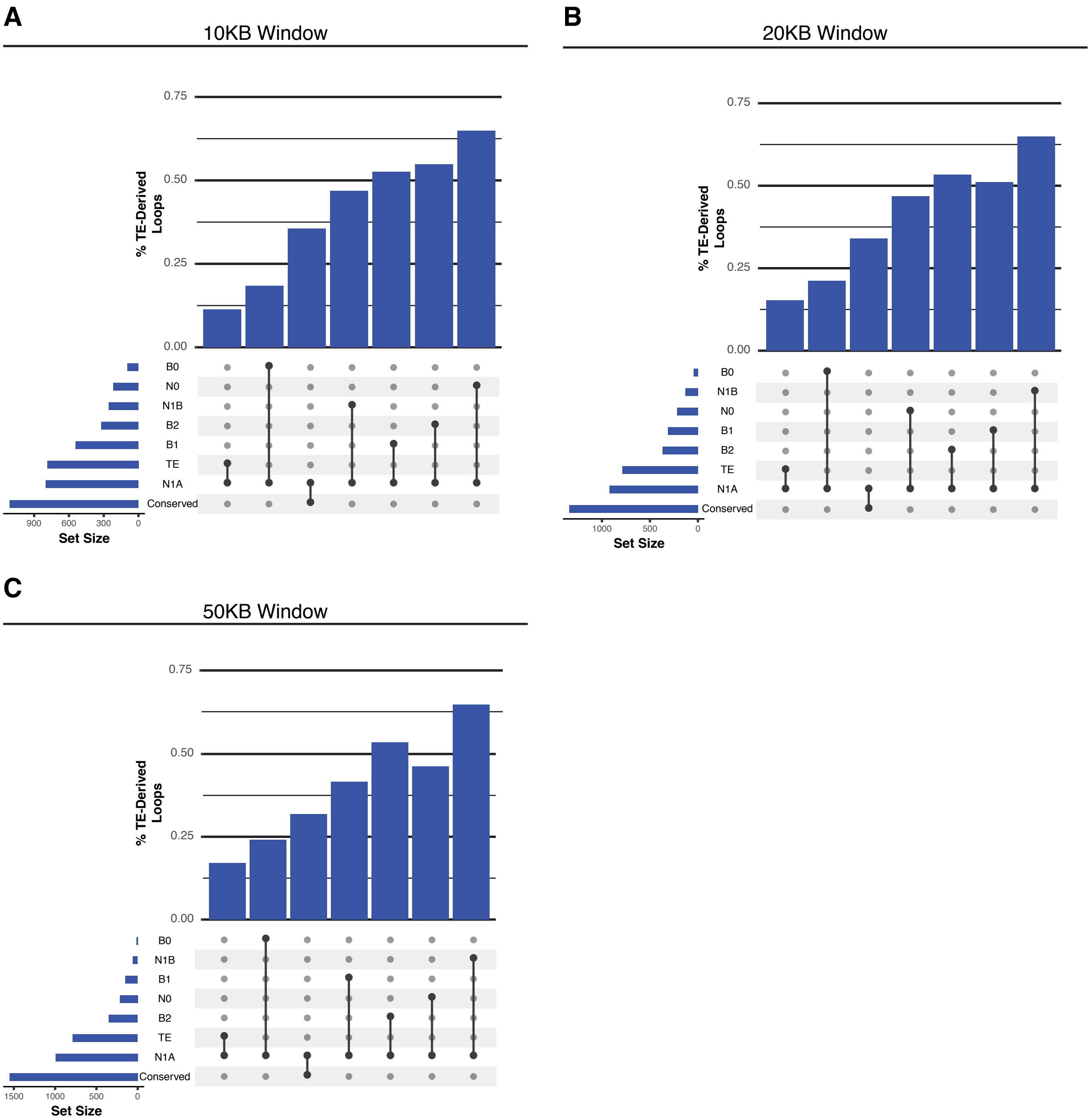

Supplementary Fig. 12: Contributions of transposable elements to conserved and non-conserved chromatin loops are not correlated with loop comparison resolution. The stringency for loops to be considered conserved was varied by extending anchor loci to windows varying from 10-50kb surrounding the center of the ChIP-seq peak annotation (See Supplementary Fig. 7). **A)** TE contributions in each conservation class with 10kb resolution. **B)** TE contributions in each conservation class with 20kb resolution. **C)** TE contributions in each conservation class with 50kb resolution.

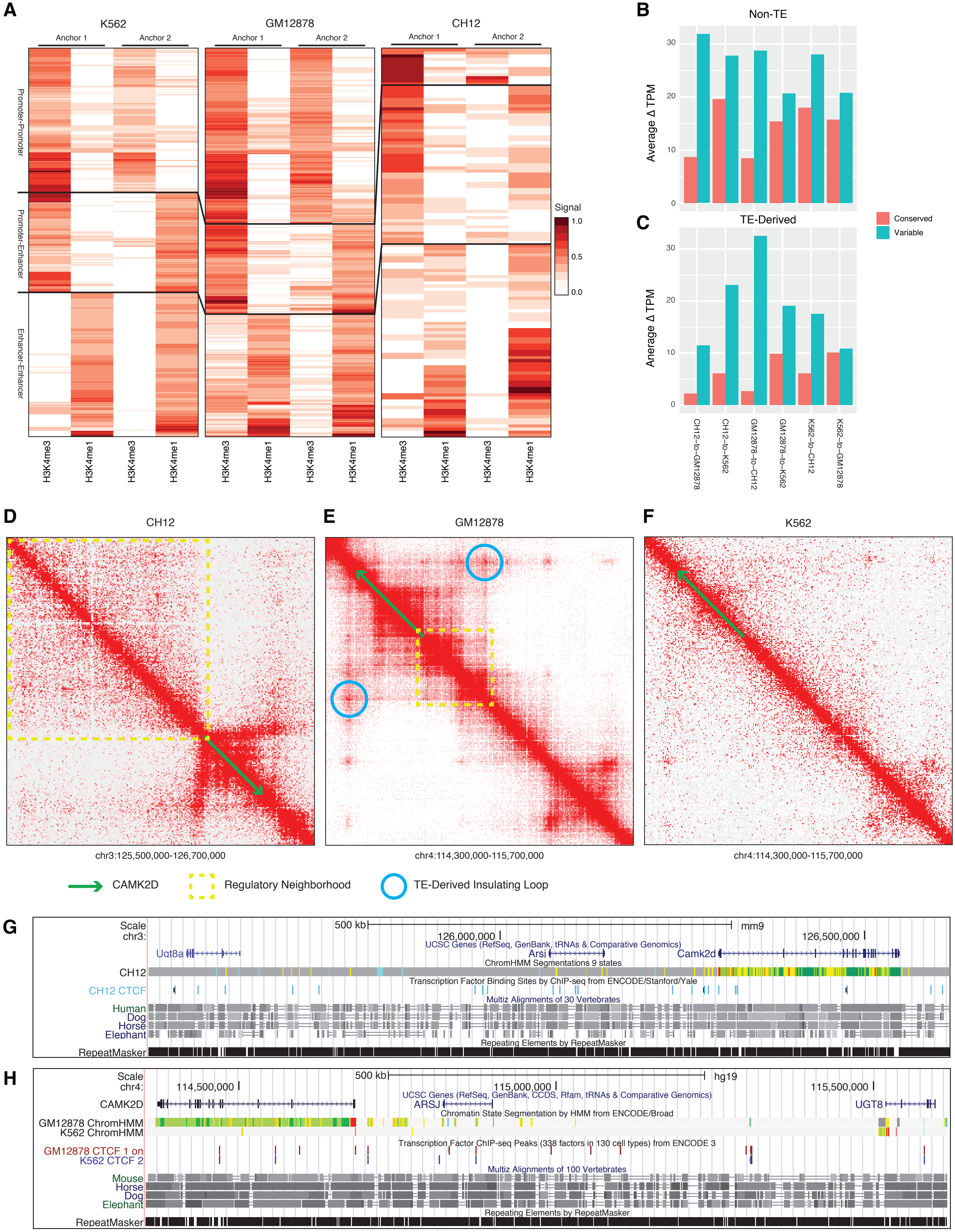
A
K562
GM12878
CH12
Anchor 1
Anchor 2
Promoter-Promoter
Promoter-Enhancer
Enhancer-Enhancer
H3K4me3
H3K4me1
Signal
1.0
0.5
0.0
B
Non-TE
Average Δ TPM
C
TE-Derived
Anerage Δ TPM
Conserved
Variable
CH12−to−GM12878
CH12−to−K562
GM12878−to−CH12
GM12878−to−K562
K562−to−CH12
K562−to−GM12878
D
CH12
chr3:125,500,000-126,700,000
E
GM12878
chr4:114,300,000-115,700,000
F
K562
chr4:114,300,000-115,700,000
CAMK2D
Regulatory Neighborhood
TE-Derived Insulating Loop
G
Scale
chr3:
500 kb
mm9
126,000,000
126,500,000
UCSC Genes (RefSeq, GenBank, tRNAs & Comparative Genomics)
Ugt8a
Arsi
Camk2d
ChromHMM Segmentations 9 states
CH12
CH12 CTCF
Transcription Factor Binding Sites by ChIP-seq from ENCODE/Stanford/Yale
Multiz Alignments of 30 Vertebrates
Human
Dog
Horse
Elephant
Repeating Elements by RepeatMasker
RepeatMasker
H
Scale
chr4:
500 kb
hg19
114,500,000
115,000,000
115,500,000
CAMK2D
UCSC Genes (RefSeq, GenBank, CCDS, Rfam, tRNAs & Comparative Genomics)
ARSJ
UGT8
Chromatin State Segmentation by HMM from ENCODE/Broad
GM12878 ChromHMM
K562 ChromHMM
Transcription Factor ChIP-seq Peaks (338 factors in 130 cell types) from ENCODE 3
GM12878 CTCF 1 on
K562 CTCF 2
Multiz Alignments of 100 Vertebrates
Mouse
Horse
Dog
Elephant
Repeating Elements by RepeatMasker
RepeatMasker

Supplementary Fig. 13: TE-derived variable chromatin loops are significantly associated with variable gene expression. A) Heat maps illustrating typical histone modification signals for H3K4me3 and H3k4me1 at loop anchors for promoter-promoter, promotoer-enhancer, and enhancer-enhancer loops in each of the three cell types in our dataset. The maximum signal value for each histone mark overlapping each loop anchor, scaled from 0-1, is plotted. **B-C)** Average gene expression differences, in units of transcripts per million (TPM), between looping datasets for all possible pairwise comparisons of cells. Loops in which one end contacts a gene promoter (i.e., is within 1kb of the TSS) and the other is distal (i.e., >= 3kb distant from its nearest TSS) were isolated from each dataset and Δ TPM (the difference in TPM between the query cell and target cell) was recorded for the gene at the promoter-proximal anchor. **B)** Average Δ TPM values for conserved and variable loops that do not include a TE-derived anchor. **C)** Average Δ TPM values for conserved and variable loops with at least one anchor derived from a TE. **D)** Hi-C plot for the TAD enclosing the CAMK2D gene (green arrow) in mouse CH12 cells. The CAMK2D regulatory neighborhood (outlined in yellow) extends from the promoter all the way to the distal TAD boundary, and is enriched throughout for interactions with the promoter. **E)** Hi-C plot showing the CAMK2D TAD in human GM12878 cells. Three CTCF-tethered loop anchors appear to act as insulators, dividing the TAD into four distinct subloops. The position of a TE-derived CTCF binding site that insulates the CAMK2D promoter from contact with the two furthest-distal loops is outlined by blue circles. The CAMK2D regulatory neighborhood is outlined in yellow outlined region. **F)** Hi-C plot showing the CAMK2D TAD in human K562 cells. The region in K562 is largely devoid of intra-TAD contacts, with the exception of a single insulater conserved with GM12878, which contacts both TAD boundaries but not the CAMK2D promoter. **G)** UCSC Genome Browser track, retrieved from the PSU mirror (https://main.genome-browser.bx.psu.edu/), for the CAMK2D TAD in mouse (mm9 assembly). The TAD contains three genes, but only CAMK2D is expressed. ChromHMM annotations for CH12 cells show the locations of regulatory features within this region, including approximately 13 annotated enhancers. The locations of CTCF binding sites in CH12 cells are also shown, as are conservation with human, dog, horse, and elephant. **H)** UCSC Genome Browser track for the CAMK2D TAD in human (hg19 assembly). The TAD contains three genes, but only CAMK2D is expressed in GM12878 cells. ChromHMM annotations for GM12878 and K562 cells show the locations of regulatory features within this region, including approximately 13 annotated enhancers in GM12878, and two in K562. The locations of CTCF binding sites in GM12878 and K562 cells are also shown, as are conservation with human, dog, horse, and elephant.
