# Supplementary material for: Transposable elements contribute to cell and species-specific chromatin looping and gene regulation in mammalian genomes
Source: Nat Commun. 2020 Apr 14;11:1796. doi: 10.1038/s41467-020-15520-5 (PMC7156512; doi:10.1038/s41467-020-15520-5)
Supplement: Supplementary file 3 — Reporting summary [file 41467_2020_15520_MOESM3_ESM.pdf]

nature research

Corresponding author(s): Alan P Boyle

Last updated by author(s): Jul 15, 2019

Reporting Summary

Nature Research wishes to improve the reproducibility of the work that we publish. This form provides structure for consistency and transparency in reporting. For further information on Nature Research policies, see Authors & Referees and the Editorial Policy Checklist .

Statistics

For all statistical analyses, confirm that the following items are present in the figure legend, table legend, main text, or Methods section.

n/a	Confirmed	
x		The exact sample size (*n*) for each experimental group/condition, given as a discrete number and unit of measurement
x		A statement on whether measurements were taken from distinct samples or whether the same sample was measured repeatedly
	x	The statistical test(s) used AND whether they are one- or two-sided<br>*Only common tests should be described solely by name; describe more complex techniques in the Methods section.*
x		A description of all covariates tested
	x	A description of any assumptions or corrections, such as tests of normality and adjustment for multiple comparisons
x		A full description of the statistical parameters including central tendency (e.g. means) or other basic estimates (e.g. regression coefficient) AND variation (e.g. standard deviation) or associated estimates of uncertainty (e.g. confidence intervals)
	x	For null hypothesis testing, the test statistic (e.g. *F*, *t*, *r*) with confidence intervals, effect sizes, degrees of freedom and *P* value noted<br>*Give P values as exact values whenever suitable.*
x		For Bayesian analysis, information on the choice of priors and Markov chain Monte Carlo settings
x		For hierarchical and complex designs, identification of the appropriate level for tests and full reporting of outcomes
x		Estimates of effect sizes (e.g. Cohen's *d*, Pearson's *r*), indicating how they were calculated

Our web collection on statistics for biologists contains articles on many of the points above.

Software and code

Policy information about availability of computer code

Data collection	No software was used for data collection.
Data analysis	bedtools v2.24.0, bx-python 0.8.1 [https://github.com/Boyle-Lab/bx-python], R 3.8.1, Kent_tools 20130806-linux.x86_64, FastQC 0.11.5, MANGO Wolverine 1.1.9 [https://github.com/adadiehl/mango], samtools 1.7, BWA 0.7.16a, mapGL.py 0.0.1 [https://github.com/adadiehl/mapGL], mapLoopLoci.py 0.0.1 [https://github.com/adadiehl/mapLoopLoci], extractAncestral.py 0.0.1 [https://github.com/adadiehl/repeatMaskerUtils], FIMO 4.10.2, score_motifs.pl 0.3 [https://github.com/adadiehl/score_motifs]

For manuscripts utilizing custom algorithms or software that are central to the research but not yet described in published literature, software must be made available to editors/reviewers. We strongly encourage code deposition in a community repository (e.g. GitHub). See the Nature Research guidelines for submitting code & software for further information.

Data

Policy information about availability of data

All manuscripts must include a data availability statement. This statement should provide the following information, where applicable:

- Accession codes, unique identifiers, or web links for publicly available datasets
- A list of figures that have associated raw data
- A description of any restrictions on data availability

The datasets supporting the conclusions of this article are included within the article and its additional files, listed in Sup. Table 1. Data processing scripts are available in the github repository [https://github.com/Boyle-Lab/TE-Driven-CTCF-Loop-Evol].

Field-specific reporting

Please select the one below that is the best fit for your research. If you are not sure, read the appropriate sections before making your selection.

☐ Life sciences ☐ Behavioural & social sciences ☒ Ecological, evolutionary & environmental sciences

For a reference copy of the document with all sections, see nature.com/documents/nr-reporting-summary-flat.pdf

Ecological, evolutionary & environmental sciences study design

All studies must disclose on these points even when the disclosure is negative.

Study description	Qualitative comparisons between human and mouse CTCF occupancy data and chromatin loop predictions were made to determine contributions of transposable element activity to conserved and divergent 3D chromatin structure. Quantitative analysis of overlap between CTCF binding sites and transposable elements (TEs) was performed to identify TE types statistically enriched for CTCF binding relative to a null hypothesis of random CTCF occupancy within TEs of all types. For permutation-based enrichment analysis, 10,000 permutations were run in order to fully sample the null distribution of CTCF occupancy within TEs, yielding a valid empirical CDF. In all analyses, Bonferroni correction was applied to p-values whenever multiple tests were performed.
Research sample	We chose two pairs of biologically-matched immune cell types from human and mouse to perform this study. These have been extensively used in comparative genomics analyses of these species, but have not been investigated in terms of CTCF-TE enrichments. All data used are listed in Supplemental Table 1.
Sampling strategy	For all analysis steps, our strategy was to utilize the most complete and up-to-date datasets available. We used all available ChIP-seq, ChIA-pet, and RNA-seq datasets for the relevant cell types with status "released" from the ENCODE DCC, and the latest annotations for repeatmasker and Hi-C data.
Data collection	All data were previously-published and obtained from public repositories, as described in Supplementary Table 1. Data collection was performed by the original authors and/or individual contributors to the ENCODE Project. Data collection protocols for ENCODE experiments are available through the ENCODE DCC and, for other datasets, through the publications referenced in Supplementary Table 1.
Timing and spatial scale	The submission dates for individual ENCODE experiments are available through the ENCODE DCC for each dataset in Supplementary Table 1. Biosample-related metadata are also available there. For other sources, we have used the most-recent and most-complete datasets available.
Data exclusions	N/A: No data were excluded from the analysis.
Reproducibility	Permutation tests were performed three times with no effect on results. For all other analyses, qualitative and deterministic measures were applied to all available data, so replication was not applicable.
Randomization	N/A: No randomized trials were performed.
Blinding	N/A: No test subjects were used so no blinding was necessary.

Did the study involve field work? ☐ Yes ☒ No

Reporting for specific materials, systems and methods

We require information from authors about some types of materials, experimental systems and methods used in many studies. Here, indicate whether each material, system or method listed is relevant to your study. If you are not sure if a list item applies to your research, read the appropriate section before selecting a response.

Materials & experimental systems

n/a	Involved in the study
☒	☐ Antibodies
☒	☐ Eukaryotic cell lines
☒	☐ Palaeontology
☒	☐ Animals and other organisms
☒	☐ Human research participants
☒	☐ Clinical data

Methods

n/a	Involved in the study
☒	☐ ChIP-seq
☒	☐ Flow cytometry
☒	☐ MRI-based neuroimaging
